# Supplementary material for: Assessing medication use patterns in patients hospitalised with COVID-19: a retrospective study
Source: BMJ Open. 2022 Dec 5;12(12):e064320. doi: 10.1136/bmjopen-2022-064320 (PMC9723413; doi:10.1136/bmjopen-2022-064320)
Supplement: Supplementary data [file bmjopen-2022-064320supp001.pdf]

Supplementary material

Table S1: Patient baseline characteristics, monotherapy by individual drug, Scotland

n (%)	Dexamethasone n=3,094	Remdesivir n=68	Tocilizumab n=145
Socio-demographics			
Median age (IQR) [years]	66 (54 – 78)	65.5 (55 – 74)	57 (45 – 66)
Age 18 – 40 [years]	342 (11.1)	5 (7.4)	31 (21.4)
Age 41 – 70 [years]	1,475 (47.7)	39 (57.4)	87 (60.0)
Age > 70 [years]	1,277 (41.3)	24 (35.3)	27 (18.6)
Sex [male]	1,659 (53.6)	40 (58.8)	91 (62.8)
Most deprived [quintile][1]	927 (30.0)	31 (45.6)	42 (29.0)
Least deprived [quintile][1]	280 (9.0)	8 (11.8)	14 (9.7)
Region: Health Board			
Ayrshire & Arran	779 (25.2)	2 (2.9)	23 (15.9)
Dumfries & Galloway	156 (5.0)	4 (5.9)	5 (3.4)
Forth Valley	656 (21.2)	1 (1.5)	19 (13.1)
Greater Glasgow & Clyde	461 (14.9)	6 (8.8)	13 (9.0)
Lanarkshire	906 (29.3)	53 (77.9)	76 (52.4)
Lothian	136 (4.4)	2 (2.9)	9 (6.2)
Vaccination status			
Unvaccinated	2,316 (74.9)	63 (92.6)	94 (64.8)
1 dose	125 (4.0)	1 (1.5)	40 (27.6)
2 doses	653 (21.1)	4 (5.9)	11 (7.6)
**Charlson score**[2]			
0	1,570 (50.7)	35 (51.5)	90 (62.1)
1 – 2	1,028 (33.2)	23 (33.8)	47 (32.4)
3 – 4	343 (11.1)	6 (8.8)	4 (2.8)
> 4	153 (4.9)	4 (5.9)	4 (2.8)
Medication at baseline: number of different items[3]			
0	294 (9.5)	4 (5.9)	34 (23.5)
1 – 4	613 (19.2)	15 (22.1)	43 (29.7)
5 – 10	1,041 (33.7)	21 (30.9)	38 (26.2)
> 10	1,146 (37.0)	28 (41.2)	30 (20.7)
Medication at baseline: specific drug classes [yes][3]			
Antihypertensive drugs	1,671 (54.0)	41 (60.3)	51 (35.2)
Antithrombotic drugs	1,093 (35.3)	22 (32.3)	36 (24.8)
Statins	1,187 (38.4)	31 (45.6)	34 (23.5)
Bronchodilator	793 (25.6)	17 (25.0)	25 (17.2)
Inhaled steroid	643 (20.8)	16 (23.5)	23 (15.9)
Diabetes medication	607 (19.6)	10 (14.7)	21 (14.5)

[1] Deprivation based on the Scottish Index of Multiple Deprivation quintiles, where 1=most deprived and 5=least deprived (25)

[2] Charlson score at baseline based on diagnoses from hospital discharge records during the 5-year period directly preceding the admission, identified using ICD-10 codes (26)

[3] Polypharmacy and medication at baseline based on prescriptions dispensed in community pharmacy during the 6-months period directly preceding the admission, identified using BNF codes (27); includes both acute and chronic medication

Table S2: Patient baseline characteristics, combination therapy, Scotland

n (%)	Dexamethasone & remdesivir n=185	Dexamethasone & tocilizumab n=530	Remdesivir & tocilizumab n=10	Dexamethasone, remdesivir & tocilizumab n=31
Socio-demographics				
Median age (IQR) [years]	65 (52 – 73.5)	58 (45 – 70)	66 (60 – 74)	56 (46 – 65)
Age 18 – 40 [years]	21 (11.4)	100 (18.8)	0	5 (16.1)
Age 41 – 70 [years]	101 (54.9)	303 (57.1)	6 (60.0)	22 (71.0)
Age > 70 [years]	62 (33.7)	128 (24.1)	4 (40.0)	4 (12.9)
Sex [male]	107 (58.2)	336 (63.3)	7 (70.0)	20 (64.5)
Most deprived [quintile][1]	57 (31.0)	156 (29.4)	3 (30.0)	11 (35.5)
Least deprived [quintile][1]	18 (9.8)	58 (10.9)	0	5 (16.3)
Region: Health Board				
Ayrshire & Arran	5 (2.7)	59 (11.1)	0	0
Dumfries & Galloway	17 (9.2)	62 (11.7)	1 (10.0)	3 (9.7)
Forth Valley	22 (12.0)	60 (11.3)	0	0
GGC	43 (23.4)	72 (13.6)	4 (40.0)	15 (48.4)
Lanarkshire	85 (46.2)	224 (42.2)	4 (40.0)	5 (16.1)
Lothian	12 (6.5)	54 (10.2)	1 (10.0)	8 (25.8)
Vaccination status				
Unvaccinated	148 (80.4)	321 (60.5)	7 (70.0)	15 (48.4)
1 dose	3 (1.6)	28 (5.3)	0	4 (12.9)
2 doses	33 (17.9)	182 (34.3)	3 (30.0)	12 (38.7)
**Charlson score**[2]				
0	96 (51.9)	348 (65.7)	7 (70.0)	13 (41.9)
1 – 2	69 (37.5)	156 (29.4)	2 (20.0)	14 (45.2)
3 – 4	12 (6.5)	17 (3.3)	0	1 (3.2)
>4	8 (4.4)	9 (1.7)	1 (10.0)	3 (9.7)
Medication at baseline: number of different items[3]				
0	15 (8.2)	97 (19.3)	2 (20.0)	1 (3.2)
1 – 4	40 (21.7)	141 (26.6)	3 (30.0)	9 (29.0)
5 – 10	60 (32.6)	162 (30.5)	3 (30.0)	14 (45.2)
> 10	69 (37.5)	131 (24.7)	2 (20.0)	7 (22.6)
Medication at baseline: specific drug classes [yes][3]				
Antihypertensive drugs	103 (56.0)	248 (46.7)	5 (50.0)	13 (41.9)
Antithrombotic drugs	57 (31.0)	129 (24.3)	4 (40.0)	7 (22.6)
Statins	74 (40.2)	173 (32.6)	4 (40.0)	8 (25.8)
Bronchodilator	57 (31.0)	85 (16.0)	2 (20.0)	5 (16.1)
Inhaled steroid	44 (23.9)	80 (15.1)	2 (20.0)	6 (19.4)
Diabetes medication	35 (19.0)	98 (18.5)	3 (30.0)	4 (12.9)

[1] Deprivation based on the Scottish Index of Multiple Deprivation quintiles, where 1=most deprived and 5=least deprived (25)

[2] Charlson score at baseline based on diagnoses from hospital discharge records during the 5-year period directly preceding the admission, identified using ICD-10 codes (26)

[3] Polypharmacy and medication at baseline based on prescriptions dispensed in community pharmacy during the 6-months period directly preceding the admission, identified using BNF codes (27); includes both acute and chronic medication

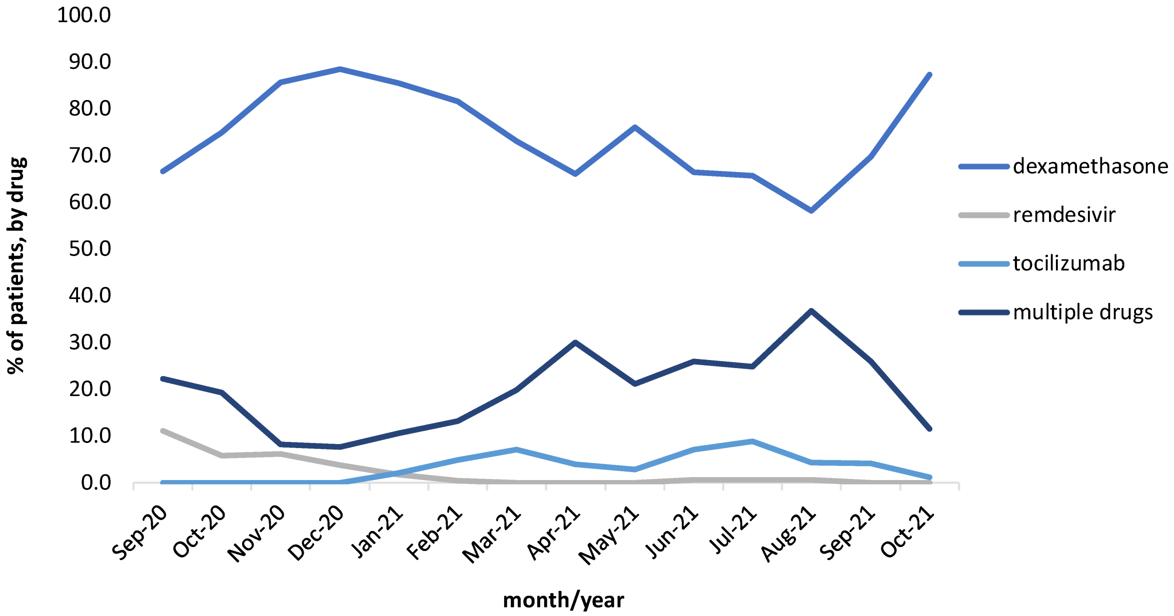

Figure S1: Percentage of patients on each treatment option over time, by month
