# Supplementary material for: Assessing medication use patterns in patients hospitalised with COVID-19: a retrospective study
Source: BMJ Open. 2022 Dec 5;12(12):e064320. doi: 10.1136/bmjopen-2022-064320 (PMC9723413; doi:10.1136/bmjopen-2022-064320)
Supplement: Author's manuscript [file bmjopen-2022-064320.draft_revisions.pdf]

BMJ Open is committed to open peer review. As part of this commitment we make the peer review history of every article we publish publicly available.

When an article is published we post the peer reviewers' comments and the authors' responses online. We also post the versions of the paper that were used during peer review. These are the versions that the peer review comments apply to.

The versions of the paper that follow are the versions that were submitted during the peer review process. They are not the versions of record or the final published versions. They should not be cited or distributed as the published version of this manuscript.

BMJ Open is an open access journal and the full, final, typeset and author-corrected version of record of the manuscript is available on our site with no access controls, subscription charges or pay-per-view fees (http://bmjopen.bmj.com).

If you have any questions on BMJ Open's open peer review process please email info.bmjopen@bmj.com

BMJ Open

Assessing medication use patterns in patients hospitalised with COVID-19: a retrospective study

Journal:	*BMJ Open*
Manuscript ID	bmjopen-2022-064320
Article Type:	Original research
Date Submitted by the Author:	06-May-2022
Complete List of Authors:	Mueller, Tanja; University of Strathclyde, Strathclyde Institute of Pharmacy and Biomedical Sciences; Public Health Scotland Glasgow Office, Clinical and Protecting Health Directorate<br>Kurdi, Amanj; University of Strathclyde, Strathclyde Institute of Pharmacy and Biomedical Sciences; Public Health Scotland Glasgow Office, Clinical and Protecting Health Directorate<br>Hall, Elliott; Public Health Scotland Glasgow Office, Clinical and Protecting Health Directorate<br>Bullard, Ian; NHS Digital<br>Wapshott, Jo; NHS Digital<br>Goodfellow, Anna; NHS Digital<br>Platt, Niketa; NHS Fife; Public Health Scotland Glasgow Office, Clinical and Protecting Health Directorate<br>Proud, Euan; Public Health Scotland Glasgow Office, Clinical and Protecting Health Directorate<br>McTaggart, Stuart; Public Health Scotland, Clinical and Protecting Health Directorate<br>Bennie, Marion; University of Strathclyde, Strathclyde Institute of Pharmacy and Biomedical Sciences; Public Health Scotland, Clinical and Protecting Health Directorate<br>Sheikh, Aziz; The University of Edinburgh Usher Institute of Population Health Sciences and Informatics; HDR UK, BREATHE Hub
Keywords:	COVID-19, THERAPEUTICS, CLINICAL PHARMACOLOGY, Health informatics < BIOTECHNOLOGY & BIOINFORMATICS

SCHOLARONE™
Manuscripts

I, the Submitting Author has the right to grant and does grant on behalf of all authors of the Work (as defined in the below author licence), an exclusive licence and/or a non-exclusive licence for contributions from authors who are: i) UK Crown employees; ii) where BMJ has agreed a CC-BY licence shall apply, and/or iii) in accordance with the terms applicable for US Federal Government officers or employees acting as part of their official duties; on a worldwide, perpetual, irrevocable, royalty-free basis to BMJ Publishing Group Ltd ("BMJ") its licensees and where the relevant Journal is co-owned by BMJ to the co-owners of the Journal, to publish the Work in this journal and any other BMJ products and to exploit all rights, as set out in our licence.

The Submitting Author accepts and understands that any supply made under these terms is made by BMJ to the Submitting Author unless you are acting as an employee on behalf of your employer or a postgraduate student of an affiliated institution which is paying any applicable article publishing charge ("APC") for Open Access articles. Where the Submitting Author wishes to make the Work available on an Open Access basis (and intends to pay the relevant APC), the terms of reuse of such Open Access shall be governed by a Creative Commons licence – details of these licences and which Creative Commons licence will apply to this Work are set out in our licence referred to above.

Other than as permitted in any relevant BMJ Author's Self Archiving Policies, I confirm this Work has not been accepted for publication elsewhere, is not being considered for publication elsewhere and does not duplicate material already published. I confirm all authors consent to publication of this Work and authorise the granting of this licence.

For peer review only

Assessing medication use patterns in patients hospitalised with COVID-19: a retrospective study

Authors: Tanja Mueller[1,2], Amanj Kurdi[1,2], Elliott Hall[2], Ian Bullard[3], Jo Wapshott[3], Anna Goodfellow[3], Niketa Platt[2,4], Euan Proud [2], Stuart McTaggart[2], Marion Bennie[1,2], Aziz Sheikh[5,6] on behalf of the EAVE II Collaboration

Affiliations: [1]Strathclyde Institute of Pharmacy and Biomedical Sciences, University of Strathclyde, Glasgow, UK; [2]Clinical and Protecting Health Directorate, Public Health Scotland, Glasgow/Edinburgh, UK; [3]NHS Digital, Leeds, UK; [4]NHS Fife, Kirkcaldy, UK; [5]Usher Institute, University of Edinburgh, Edinburgh, UK; [6]HDR UK BREATHE Hub, Edinburgh, UK

Corresponding author:

Tanja Mueller, Strathclyde Institute of Pharmacy and Biomedical Sciences, University of Strathclyde, 161 Cathedral Street, Glasgow G4 0RE, UK; email tanja.muller@strath.ac.uk

ORCID ID:

Tanja Mueller: 0000-0002-0418-4789
Amanj Kurdi: 0000-0001-5036-1988
Elliott Hall: 0000-0003-0540-0735
Stuart McTaggart: 0000-0001-6060-9019
Marion Bennie: 0000-0002-4046-629x
Aziz Sheikh: 0000-0001-7022-3056

Keywords: drug utilisation, COVID-19, in-hospital treatment, electronic prescribing

Word count: abstract: 275; main text: 3,671 (excluding tables, figures, references)

Table/figure count: 2 tables, 1 figure, 3 online supplements comprising 3 figures and 4 tables

For peer review only

Abstract

Objective: To describe patterns of medication use – i.e. dexamethasone; remdesivir; and tocilizumab – in the management of hospitalised COVID-19 patients.

Design and setting: Retrospective observational study, using routinely collected, linked electronic data from clinical practice in Scotland. Data on drug exposure in secondary care has been obtained from the Hospital Electronic Prescribing and Medicines Administration System.

Participants: Patients being treated with the drugs of interest and hospitalised for COVID-19 between 1st March 2020 and 10th November 2021.

Outcomes: Identification of patients subject to the treatments of interest; summary of patients' baseline characteristics; description of medication use patterns and treatment episodes. Analyses were descriptive in nature.

Results: Overall, 4063 patients matching the inclusion criteria were identified in Scotland, with a median (IQR) age of 64 years (52 – 76). Among all patients, 81.4% (n=3307) and 17.8% (n=725) were treated with one or two medicines, respectively; dexamethasone monotherapy accounted for the majority (n=3094, 76.2%) followed by dexamethasone in combination with tocilizumab (n=530, 13.0%). Treatment patterns were variable over time but roughly followed the waves of COVID-19 infections; however, the different drugs were used to varying degrees during the study period.

The median (IQR) treatment duration differed by medicine: dexamethasone 5 days (2 – 9); remdesivir 5 days (2 – 5); and tocilizumab 1 day (1 – 1). The overall median (IQR) length of hospital stay among all patients included in the study cohort was 9 days (5 – 17); 24.7% of patients died in hospital.

Conclusion: The use of adjuvant medicines in hospitalised COVID-19 patients appears in line with evolving evidence and changing treatment guidelines. In-hospital electronic prescribing systems are a valuable source of information, providing detailed patient-level data on in-hospital drug use.

For peer review only - http://bmjopen.bmj.com/site/about/guidelines.xhtml

ARTICLE SUMMARY

Strengths and limitations of this study:

- This is the first study to use hospital electronic prescribing data to assess patient level, in-hospital medicines use in the UK.
- The availability of in-hospital prescribing data and the opportunity to deterministically link various datasets enabled an in-depth description of the use of repurposed drugs in patients hospitalised with COVID-19.
- Due to the observational nature of this study and limitations on the data, analyses of clinical safety or effectiveness of the drugs of interest were not feasible.

For peer review only

INTRODUCTION

The new "severe acute respiratory syndrome coronavirus 2" (SARS-CoV-2) has spread rapidly since its emergence in China in late 2019, and the disease caused by it – known as COVID-19 – is impacting population health globally (1). Although symptoms and disease severity may vary, COVID-19 primarily affects the respiratory system, resulting in breathlessness and, potentially, hospitalisation; in severe cases, patients require intensive care including mechanical ventilation (2). Further complications including arterial and venous thromboembolism are ascribed to the pro-thrombotic nature of the disease (3).

Even though vaccines have rapidly been developed and immunisation campaigns were rolled-out at speed in many countries including the UK, the search for effective treatments is ongoing due to limits on vaccine effectiveness (4), immune escape particularly among Variants of Concern (VoC) (5), and waning immunity following both infection and vaccination (6). While a range of diverse options were discussed early in the pandemic – spanning from antibiotics to convalescent plasma – only a small number of medicines were found to be effective in clinical trials (7), and subsequently approved for use by regulators. Medicines recommended for use in patients hospitalised with COVID-19 in the UK comprise remdesivir, an antiviral drug (introduced in guidelines 29th May 2020); steroids, most prominently dexamethasone (16th June 2020); and monoclonal antibodies including tocilizumab (8th January 2021) (8–10). Most recently, a number of antivirals and monoclonal antibodies have also been approved for use in the community, including, e.g., molnupiravir and sotrovimab (11).

There is a need for real-world studies assessing the uptake, effectiveness, and safety of these drugs to complement the findings from rapidly undertaken efficacy trials. Nevertheless, thus far, evidence from clinical practice is limited; in particular, there are very few publications offering detailed descriptions of in-hospital drug use. While a range of studies have investigated the use of repurposed and adjuvant drugs in COVID-19 patients, the majority of publications have focused on the effect of specific drugs on disease outcomes rather than the drug use itself (e.g., (12–14)). Consequently, studies rarely provide in-depth information on treatment patterns (including temporal and geographical

For peer review only - http://bmjopen.bmj.com/site/about/guidelines.xhtml

variation) or details on duration of treatment – potentially compounded by the unavailability of in-depth, patient-level information (15–17).

In the UK, the drive towards digitisation in health care has resulted in the implementation of electronic prescribing; initially in primary care, but subsequently also in specialist settings (e.g., oncology) and secondary care, thereby facilitating data sharing across settings (18). In Scotland, the roll-out of the Hospital Electronic Prescribing and Medicines Administration (HEPMA) system was initiated in 2014 in line with the Scottish eHealth strategy (19,20), and HEPMA is now available in hospitals across six of the 14 regional organisations (Health Boards) within NHS Scotland tasked with planning and delivering services to their local populations. Similarly, implementation of the Electronic Prescribing and Medicines Administration (EPMA) system has started in secondary care NHS Trusts in England, where central data collection for COVID-19 purposes currently covers around 9% of the total prescribing activity in secondary care (21).

The aim of this study was to investigate the use of in-hospital medication for the treatment of COVID-19, namely dexamethasone, remdesivir and tocilizumab. Our specific objectives were: to describe medicines use over time and across geographical areas; to summarise patients' baseline characteristics; and to describe treatment patterns as well as hospital admission episodes, including their outcomes.

METHODS

Study design and setting

The study was designed as a retrospective cohort study using routinely collected linked health care data, covering the time period from 1st March 2020 to 10th November 2021 based on the timeline of events (World Health Organization interim guidance/declaration of the pandemic) and data availability. Health care throughout the UK is provided via the tax-funded National Health Services (NHS), with services being offered primarily without payment at the point of care; nevertheless, there are differences between NHS Scotland and NHS England, both being separate organisations with unique structures and processes in place (22,23).

For peer review only - http://bmjopen.bmj.com/site/about/guidelines.xhtml

The main study setting was Scotland, where every resident is allocated a unique patient identifier (the Community Health Index (CHI) number) the use of which is mandatory throughout the health and care system (24), thereby enabling deterministic linkage of electronic patient records. Since implementation of technical solutions takes place at Health Board level and regional time frames depend on local requirements and circumstances, at the time of study conduct, HEPMA had been implemented fully or partially in six Health Boards: NHS Ayrshire & Arran; NHS Dumfries & Galloway; NHS Forth Valley; NHS Greater Glasgow & Clyde (GGC); NHS Lanarkshire; and NHS Lothian, covering an estimated 3.6M people or approximately 65% of the Scottish population (5.4M) (25). However, as the HEPMA implementation started at different points in time in the various Health Boards and roll-out across hospital sites was incremental, data coverage was incomplete for two Health Boards: NHS GGC, where implementation started in late 2020, i.e. after the start of the study period; and NHS Lothian, where very few hospitals were initially included. In addition, coverage within individual hospitals differed due to the use of existing systems instead of (or in addition to) HEPMA in certain areas, e.g. in intensive care units (ICUs).

Data sources

Briefly, HEPMA was used to extract any prescriptions of the medications of interest. Using CHI numbers, HEPMA was subsequently linked to the Scottish Morbidity Records inpatient dataset (SMR01) and the Electronic Communication of Surveillance Scotland (ECOSS) data to identify the study cohort and ensure that the medications of interest were administered during a COVID-19 related hospital admission episode. A number of other datasets (including, but not limited to, primary care prescriptions and death records) were also available through the Early Pandemic Evaluation and Enhanced Surveillance of COVID-19 (EAVE II) surveillance platform (26). Further details can be found in the protocol (27).

Study population

With drug use being the specific focus of the study, the main study cohort comprised patients who had received at least one of the medications of interest (i.e., dexamethasone, remdesivir, tocilizumab) while being admitted to hospital in the aforementioned Scottish Health Boards between 1st March 2020 and 10th November 2021 for suspected or confirmed

For peer review only - http://bmjopen.bmj.com/site/about/guidelines.xhtml

COVID-19. Hospitalisations for COVID-19 were defined as admissions within 28 days of a positive reverse transcriptase polymerase chain reaction (RT-PCR) test and/or with an International Classification of Diseases, 10th edition (ICD-10) code for COVID-19 (U07.1, U07.2) recorded on a patient's hospital admission file as the primary or secondary diagnosis.

Study outcomes, covariates, and statistical analyses

The primary outcome was observed treatment patterns among patients with any of the medications of interest over the duration of the study period. In addition, secondary outcomes included summaries of exposure in terms of administered dose and duration of treatment; as well as length of hospital stay and in-hospital mortality.

Patient characteristics, including socio-demographic factors, COVID-19 vaccination status, disease-related aspects, comorbidities, and concomitant medication, were summarised at baseline. Detailed definitions, descriptions, and relevant data sources can be found in the protocol (27).

All analyses were descriptive in nature; results were expressed as counts/frequencies for categorical variables, and median/inter-quartile range (IQR) for continuous variables. Analyses were conducted using R/R Studio V.3.6.1, and Stata 15.

Partial replication of analyses in England

The analyses were partially replicated in England using readily available data to evaluate the feasibility of conducting UK-wide observational studies using in-hospital prescribing systems, with linkage based on NHS number and verified using the patient demographic system (28). Data availability was more restricted than in Scotland: EPMA data were used to identify patients prescribed any of the medicines of interest; patients in EPMA were then linked to Hospital Episode Statistics (HES) data (29) to identify patients who had been admitted to hospital with a diagnosis of COVID-19. Further details, such as information regarding COVID-19 testing, vaccination status, or additional details on hospital admissions, were unavailable for the purpose of this study at the time of study conduct.

For peer review only

For peer review only - http://bmjopen.bmj.com/site/about/guidelines.xhtml

Similar to the main cohort, the English cohort comprised patients who received any of the medicines of interest while being admitted to one of the hospitals submitting EPMA data between 1st March 2020 and 10th November 2021 for COVID-19. Hospitalisations for COVID-19 were defined as episodes with an ICD-10 code for COVID-19 (U07.1, U07.2) as primary or secondary diagnosis. Obtainable outcomes included observed treatment patterns in England during the study period; and a limited set of baseline patient characteristics.

Patient and public involvement

The EAVE II Public Advisory group are a diverse group of PPI contributors who meet monthly to incorporate the views of patients and the public into research using the EAVE II dataset. This includes shaping of research via the EAVE II Steering Group, which is attended by two lay leads. A lay summary, co-written by our PPI contributors, will be shared via the outputs section of the EAVE II website, hosted by the University of Edinburgh (26).

Ethical approval

Ethical and information governance approvals have been obtained by the National Research Ethics Service Committee (REC), South East Scotland 02 (REC number: 12/SS/0102) and the Public Benefit and Privacy Panel for Health and Social Care (reference number: 1920-0279), respectively.

Reporting of the study follows the STROBE (Strengthening the Reporting of Observational Studies in Epidemiology) guidelines (30); a STROBE checklist can be found in the supplementary material.

RESULTS

At the study locations in Scotland, 4063 patients admitted to hospital with a suspected or confirmed diagnosis of COVID-19 received at least one of the drugs of interest during the study period. The largest group of patients was treated in NHS Lanarkshire (n=1353, 33.3%), followed by NHS Ayrshire & Arran (n=868, 21.4%); NHS Forth Valley (n=758, 18.7%); and NHS GGC (n=614, 15.1%). The remaining two Health Boards – NHS Dumfries & Galloway and

For peer review only - http://bmjopen.bmj.com/site/about/guidelines.xhtml

For peer review only

NHS Lothian – accounted for relatively small patient populations (n=248 (6.1%) and n=222 (5.5%), respectively).

Patient baseline characteristics

The median age of all patients at the time of hospital admission was 64 years (IQR 52 – 76), with 37.6% of patients over 70 years of age. Just over half of the patients (55.6%) were male. Polypharmacy – i.e. being prescribed five or more different medications simultaneously, measured during the six months directly preceding hospital admission – was observed among 2752 patients (67.7%); the most commonly prescribed medication included antihypertensive drugs, statins, and antithrombotic drugs. See table 1 for details, and tables S1 and S2 in the supplementary material for a breakdown by individual drug.

Table 1: Patient baseline characteristics in Scotland

n (%)	Scotland n=4,063
Socio-demographics	
Median age (IQR) [years]	64 (52 – 76)
Age 18 – 40 [years]	504 (12.4)
Age 41 – 70 [years]	2,033 (50.0)
Age > 70 [years]	1,526 (37.6)
Sex [male]	2,260 (55.6)
Most deprived [quintile][1]	1,227 (30.2)
Least deprived [quintile][1]	383 (9.4)
Vaccination status	
Unvaccinated	2,964 (73.0)
1 dose	172 (4.2)
2 doses	927 (22.8)
Medication at baseline: number of different items[2]	
0	447 (11.0)
1 – 4	864 (21.3)
5 – 10	1,339 (33.0)
> 10	1,413 (34.8)
Medication at baseline: specific drug classes [yes][2]	
Antihypertensive drugs	2,141 (52.7%)
Antithrombotic drugs	1,348 (33.2%)
Statins	1,511 (37.2%)
Bronchodilator	984 (24.2%)
Inhaled steroid	814 (20.0%)
Diabetes medication	778 (19.2%)

[1] Deprivation based on the Scottish Index of Multiple Deprivation quintiles, where 1=most deprived and 5=least deprived (31)
[2] Polypharmacy and medication at baseline based on prescriptions dispensed in community pharmacy during the 6-months period directly preceding the admission, identified using BNF codes (32); includes both acute and chronic medication

Medication use patterns

For peer review only - http://bmjopen.bmj.com/site/about/guidelines.xhtml

Treatment with dexamethasone, remdesivir, and tocilizumab in patients with COVID-19 was variable over time. Generally speaking, prescribing patterns roughly followed the waves of COVID-19 infections; however, the different medicines were used to varying degrees and at diverging points in time during the study period. While dexamethasone use was observed throughout this period, remdesivir was primarily prescribed in 2020, whereas routine tocilizumab use started in early 2021 (see also figure 1, and figure S1 in the supplementary material for a presentation of confirmed cases in Scotland during the study period (33)).

[figure 1]

Furthermore, although the majority of patients were treated with one drug only (n=3307, 81.4%), some patients received two (n=725, 17.8%) or all three (n=31, 0.8%). Most of the patients treated with more than one drug received dexamethasone and tocilizumab in combination (n=530/756, 70.1%); nevertheless, overall, the timing/sequencing of the drugs given was very diverse, with no clear patterns emerging among the patients who received two or more of them. In addition, the use of multiple drugs increased over time. For details, see table 2.

Table 2: Patterns of drug use in Scotland between 1st March 2020 and 10th November 2021

n (%)	Overall[1]	Dexamethasone[2]	Remdesivir[2]	Tocilizumab[2]	More than one drug[3]
Scotland					
Total	4,063	3,094 (76.2)	68 (1.7)	145 (3.6)	756 (18.5)
Mar – Jun 2020	6	6 (100)	0	0	0
Jul – Sep 2020	31	21 (67.7)	3 (9.7)	0	7 (22.6)
Oct – Dec 2020	860	711 (82.7)	45 (5.2)	0	104 (12.1)
Jan – Mar 2021	1,461	1,203 (82.3)	15 (1.0)	55 (3.8)	188 (12.9)
Apr – June 2021	290	199 (68.8)	1 (0.3)	16 (5.5)	74 (25.5)
Jul – Sep 2021	1,324	874 (66.0)	4 (0.3)	73 (5.5)	373 (28.2)
Oct – Nov 2021[4]	91	80 (87.9)	0	1 (1.1)	10 (11.0)

[1] Includes all patients treated with at least one of the drugs of interest during the study period; dates of inclusion of each drug in NHS treatment guidelines: remdesivir 29 May 2020; dexamethasone 16 June 2020; tocilizumab 8 January 2021 (8–10)
[2] includes only patients treated exclusively with one of the drugs of interest
[3] includes patients treated with two or more of the drugs of interest, in any combination/sequence
[4] includes patients admitted to hospital up to and including 10th November, 2021

Hospital admission and treatment episodes

Of the Scottish study cohort, 3745 patients (92.2%) had COVID-19 recorded as their primary diagnosis for hospital admission; 109 patients (2.7%) did not have COVID-19 as a diagnosis

For peer review only - http://bmjopen.bmj.com/site/about/guidelines.xhtml

on their admission records and were identified based on a positive RT-PCR test alone. The vast majority (n=3913, 96.8%) of admissions were emergency admissions, with the majority admitted via the Acute Assessment Unit significant care facility (n=2157/4,063, 53.1%).

Patients usually started treatment very soon after admission, with a median time between hospital admission and treatment initiation of 1 day (IQR 1 – 2) across the entire Scottish cohort; there was very little difference with regards to treatment initiation between the individual drugs or any of the combinations thereof. The median treatment duration overall was 4 days (IQR 1 – 8) but this differed by drug and whether patients were treated with one drug only, or a combination of drugs: individually, dexamethasone and remdesivir were given for a median of 5 days (IQR 2 – 8 and 3 – 5, respectively); in contrast, among patients treated with dexamethasone and one other drug (either remdesivir or tocilizumab), median duration of dexamethasone treatment was 7 days (IQR 4 – 9). Tocilizumab was given as a single dose only (median 1 day, IQR 1 – 1) regardless of treatment modality.

While standard dosing regimens were in place for both dexamethasone and remdesivir, tocilizumab is dosed based on patient weight, which was reflected in the observed first/daily and overall doses given to patients. For dexamethasone, the median dose was 6mg once a day (IQR 6 – 6), with a median total dose of 30mg (IQR 12 – 48); however, the median total dose was lower in patients treated exclusively with dexamethasone (24mg, IQR 12 – 48) in line with the shorter duration of treatment in comparison to patients treated with a combination of drugs. For remdesivir, the starting dose was 200mg at day one followed by 100mg once daily on subsequent days for the vast majority of patients, with an overall median total dose of 600mg (IQR 300 – 600). The median dose of tocilizumab given to patients in Scotland was 800mg (IQR 600 – 800).

The overall median length of hospital stay of all patients included in the study cohort, regardless of whether patients were discharged or died, was 9 days (IQR 5 – 17), with minor differences between patients being treated with dexamethasone (median 8 days, IQR 5 – 17); remdesivir (median 11 days, IQR 8 – 18); or tocilizumab (median 13 days, IQR 8 – 24) as a single agent. The majority of patients (2807, 69.1%) were discharged to a private residence, whereas 1002 patients (24.7%) died in hospital.

For peer review only - http://bmjopen.bmj.com/site/about/guidelines.xhtml

Partial replication of analyses in England

In England, a total of 7680 COVID-19 patients admitted to one of the included hospitals were treated with at least one of the drugs of interest. The median age was 68 years (IQR 55 – 81), and 46.8% of patients were over 70 years old; the share of male patients was slightly higher than in Scotland with 57.0%. The vast majority of patients were treated with one drug only (n=7191, 93.6%), primarily dexamethasone. Nevertheless, some patients received two (n=469, 6.1%) or all three (n=20, 0.3%), and the most common combination in England was dexamethasone and remdesivir (n=270/489, 55.2%). Further details are presented in the supplementary material; patient baseline characteristics can be found in table S3 (34), and an overview of prescribing patterns over time is presented in table S4 and figure S2.

DISCUSSION

Using routinely collected data from clinical practice, our findings demonstrate changes in the use of specific drugs over time, aligned with waves of COVID-19 infections and changing treatment guidelines. Whereas dexamethasone was used throughout the study period, remdesivir was primarily prescribed in 2020, whilst routine tocilizumab use started in early 2021. Furthermore, in Scotland, 18.5% of patients were treated with varying drug combinations. This is, to our knowledge, one of the first studies to provide detailed descriptions of both patient characteristics as well as in-hospital treatment patterns for all three drugs of interest, covering a relatively large cohort of patients hospitalised with COVID-19.

In Scotland, the majority of patients being treated with any of the three drugs of interest while admitted to hospital for COVID-19, as captured by the available in-hospital prescribing data, were elderly, with a median age of 64 years; furthermore, prescribing data from community care indicated that patients had a range of existing comorbidities, including diabetes, asthma/COPD, hypertension, and possibly other cardiovascular diseases. Although the exact indications for received concomitant medication is not known as this information is not captured within the prescribing database used in Scotland, this is in line with expectations based on existing evidence, as disease severity – and, consequently, hospitalisation rates and/or disease outcomes – were shown to be associated with patient

For peer review only - http://bmjopen.bmj.com/site/about/guidelines.xhtml

age and the presence of certain conditions (35). Since drug use is closely linked with age (generally speaking, the older a patient, the more medications they will be on) the high level of polypharmacy, i.e. taking five or more different drugs simultaneously, observed among the study cohort was not entirely unexpected, even though the extent of excessive polypharmacy – i.e. 10 or more different drugs – was somewhat surprising; this might, however, have been influenced by the method of analysis, as the number of drugs prescribed to patients reflects both chronic and acute medication and could have been inflated by not accounting for treatment changes (e.g. patients switching from one anti-hypertensive drug to another during the assessed time period).

As an aside, while the majority of patients captured in this study (73.0%) were unvaccinated, this might primarily be ascribable to the timing of the study, since a large part of the cohort stems from before the vaccination campaign was rolled-out across Scotland. Vaccinations, initially among NHS and care home staff and care home residents, commenced in 8th December 2020, and were subsequently offered in stages; first to clinically vulnerable populations and the elderly, before being available to all adult residents and, subsequently, children aged 5 years and above (36).

Of the three drugs of interest, dexamethasone was most widely used in hospitalised COVID-19 patients; while 76.2% of patients in the Scottish cohort received this steroid, only 1.7% and 3.6% of patients were treated with remdesivir and tocilizumab, respectively. Dexamethasone was used through the study period, with two prominent peaks: first in January 2021, and then again in September 2021 – in line with COVID-19 waves in the UK. In contrast, remdesivir was prescribed early on (it was introduced in guidelines in late May 2020), but its use decreased soon after in Scotland, being replaced by tocilizumab from January 2021 onwards. Observed changes in treatment pattern over time are, however, in line with evolving knowledge and, consequently, changes in treatment guidelines (8–10). Interestingly, the number of patients receiving more than one of the studied drugs – most prominently, dexamethasone and tocilizumab – increased over time; anecdotally, the addition of dexamethasone to tocilizumab treatment has been described in other observational studies (e.g. (14)). Since there was wide variety in both the combination and sequencing of drugs and treatment decisions might have been influenced by a number of

For peer review only - http://bmjopen.bmj.com/site/about/guidelines.xhtml

factors, including disease severity and other, unknown patient-related aspects, interpretation of these findings is very difficult without further information and would, at this point, be merely speculative.

Duration of treatment was mostly in line with guidelines, with a median of five days for remdesivir and a single dose of tocilizumab; similar findings regarding remdesivir have been described in a study conducted in Hong Kong (37). However, duration of dexamethasone treatment appeared shorter than recommended, with a median of five instead of ten days – albeit with a rather wide confidence interval of two to nine days. Reasons for this are unclear, but findings could potentially be related to patients being moved to a different ward with no HEPMA coverage (e.g. ICU due to deterioration of condition); or being discharged. Alternatively, patients might indeed have stopped treatment with dexamethasone earlier than recommended. Unfortunately, reasons for neither initiating nor discontinuing treatment are recorded on HEPMA.

When interpreting results, two further relevant features of prescribing practices need to be taken into account. First, dexamethasone is not the only steroid to be used in patients with COVID-19 (prednisolone is, e.g., used in pregnant patients) and, due to intermittent drug shortages, tocilizumab has on occasion been replaced by sarilumab (amongst others). As these alternatives were not included in the analyses, total drug use may have been underestimated within the cohort. Second, the three drugs of interest are given to different groups of patients, at different points in time on their disease journey. Accordingly, direct comparison between the use of the different drugs, the patients receiving them, and – most importantly – observed patient outcomes, may not be particularly meaningful without considering the complexity of cases and possible variations in treatment based on patient, prescriber, and/or wider contextual aspects.

Study limitations

Although the use of HEPMA/EPMA offers unprecedented opportunities to conduct drug utilisation research within a secondary care setting, this study also has a number of limitations, which need to be considered when interpreting findings – most importantly, data were incomplete for both Scotland and England. In Scotland, data coverage was

For peer review only

For peer review only - http://bmjopen.bmj.com/site/about/guidelines.xhtml

variable between as well as within Health Boards. Some of the major hospitals in NHS Lothian were, for instance, not captured, and data from ICUs were missing as these do not routinely use HEPMA. Similarly, use of the EPMA system in England varies by Trust and may not include settings such as ICUs. Furthermore, the number of hospitals included in current data provisioning agreements is limited. This may have affected the accuracy of findings; for instance, the number of patients treated with the medicines of interest – in particular, tocilizumab – might have been underestimated due to its use in ICUs. In addition, there are implications on the comparability of findings. Direct comparison of treatment patterns and patient characteristics across Scottish Health Boards, for example, is inadvisable at this point due to the incremental roll-out of the system, and this is even more true for comparisons between Scotland and England as population coverage and the availability of datasets beyond HEPMA/hospital episode records differed considerably. Finally, caution is advised when interpreting patient characteristics. This study presents a snapshot of hospitalised COVID-19 patients, describing only a sample of patients admitted to hospital who started treatment with any of the drugs of interest in areas where HEPMA/EPMA has been implemented. Consequently, these characteristics should not be considered representative of all hospitalised COVID-19 patients in Scotland and/or England.

CONCLUSION

In conclusion, this study confirms the value and usefulness of using HEPMA/EPMA data for research, while also highlighting its limitations. HEPMA/EPMA offers the opportunity to obtain previously unavailable patient-level information regarding in-hospital drug treatment; however, the data is not intuitive and requires multi-disciplinary expertise. In addition, linkage with other datasets is likely required depending on the research question to be answered.

Author contributions: TM, AK, SM, MB conceptualised and designed the study. TM, AK, EH curated the Scottish data and performed the analyses. IB, JW, AG curated the English data and performed the analyses. NK, IP, SM, MB, AS contributed to data interpretation. MB and AS had the clinical and academic oversight of the project. All authors have contributed to drafting the paper or revising it critically and have approved of the final version.

For peer review only - http://bmjopen.bmj.com/site/about/guidelines.xhtml

EAVE II Collaboration: Colin R Simpson, School of Health, Victoria University of Wellington, Wellington, New Zealand and Usher Institute, University of Edinburgh, Edinburgh, UK; Holly Tibble, Usher Institute, University of Edinburgh, Edinburgh, UK; Chris Robertson, Public Health Scotland, Glasgow, UK and Department of Mathematics and Statistics, University of Strathclyde, UK; critically reviewed the study proposal and the draft manuscript.

Acknowledgements: We thank Wendy Inglis-Humphrey and Vicky Hammersley for their support with project management and administration. This work is only possible because of the wealth of information collected by the NHS as part of routine care.

Funding information: This study is part of the EAVE II project. EAVE II is funded by the Medical Research Council [MC_PC_19075] with the support of BREATHE – The Health Data Research Hub for Respiratory Health [MC_PC_19004], which in turn is funded through the UK Research and Innovation Industrial Strategy Challenge Fund and delivered through Health Data Research UK. Additional support has been provided through the Scottish Government DG Health and Social Care and the Chief Scientist's Office of the Scottish Government (no grant/award number).

Competing interests: AS reports grants from NIHR, MRC, and HDR-UK; and MB, AK, and TM report grants from HDR-UK during the conduct of this study. All other authors report no potential conflicts of interest.

Data availability statement: NHS data are confidential and only available upon request subject to approval by a Caldicott Guardian/the Public Benefit and Privacy Panel for Health and Social Care in Scotland, and the Data Access Request Service in England.

Patient consent for publication: Not required.

Ethics approval statement: Ethical and information governance approvals have been obtained by the National Research Ethics Service Committee (REC), South East Scotland 02 (REC number: 12/SS/0102) and the Public Benefit and Privacy Panel for Health and Social Care (reference number: 1920-0279), respectively.

For peer review only

For peer review only - http://bmjopen.bmj.com/site/about/guidelines.xhtml

References

1. WHO. Coronavirus disease (COVID-19) – World Health Organization [Internet]. 2022 [cited 2022 Feb 15]. Available from: https://www.who.int/emergencies/diseases/novel-coronavirus-2019

2. CDC. Coronavirus Disease 2019 (COVID-19) – Symptoms [Internet]. Centers for Disease Control and Prevention. 2021 [cited 2022 Feb 15]. Available from: https://www.cdc.gov/coronavirus/2019-ncov/symptoms-testing/symptoms.html

3. Chen A tian, Wang C yu, Zhu W ling, Chen W. Coagulation Disorders and Thrombosis in COVID-19 Patients and a Possible Mechanism Involving Endothelial Cells: A Review. Aging Dis. 2022 Feb 1;13(1):144–56.

4. Institute for Health Metrics and Evaluation. COVID-19 vaccine efficacy summary [Internet]. Institute for Health Metrics and Evaluation. 2022 [cited 2022 Feb 25]. Available from: https://www.healthdata.org/covid/covid-19-vaccine-efficacy-summary

5. Hu J, Peng P, Cao X, Wu K, Chen J, Wang K, et al. Increased immune escape of the new SARS-CoV-2 variant of concern Omicron. Cell Mol Immunol. 2022 Feb;19(2):293–5.

6. Katikireddi SV, Cerqueira-Silva T, Vasileiou E, Robertson C, Amele S, Pan J, et al. Two-dose ChAdOx1 nCoV-19 vaccine protection against COVID-19 hospital admissions and deaths over time: a retrospective, population-based cohort study in Scotland and Brazil. The Lancet. 2022 Jan 1;399(10319):25–35.

7. Nuffield Department of Population Health. RECOVERY - Randomised Evaluation of COVID-19 Therapy [Internet]. 2022 [cited 2022 Feb 15]. Available from: https://www.recoverytrial.net/

8. Scottish Government. COVID-19 therapeutic alert: Dexamethasone in the treatment of COVID-19: implementation and management of supply for treatment in hospitals [Internet]. [cited 2021 Aug 6] p. 5. Available from: https://www.sehd.scot.nhs.uk/publications/DC20200616COVID-19Dexamethasone.pdf

9. NHS England. Coronavirus » Interim Clinical Commissioning Policy: Remdesivir for patients hospitalised with COVID-19 (adults and children 12 years and older) [Internet]. [cited 2021 Mar 23]. Available from: https://www.england.nhs.uk/coronavirus/publication/interim-clinical-commissioning-policy-remdesivir-for-patients-hospitalised-with-covid-19-adults-and-children-12-years-and-older/

10. NHS England. Coronavirus » Interim Clinical Commissioning Policy: Tocilizumab for hospitalised patients with COVID-19 pneumonia (adults) [Internet]. [cited 2021 Mar 23]. Available from: https://www.england.nhs.uk/coronavirus/publication/interim-clinical-commissioning-policy-tocilizumab-for-hospitalised-patients-patients-with-covid-19-pneumonia-adults/

For peer review only - http://bmjopen.bmj.com/site/about/guidelines.xhtml

11. Department of Health & Social Care. Coronavirus » Interim clinical commissioning policy: neutralising monoclonal antibodies or antivirals for non-hospitalised patients with COVID-19 [Internet]. 2022 [cited 2022 Feb 20]. Available from: https://www.england.nhs.uk/coronavirus/publication/interim-clinical-commissioning-policy-neutralising-monoclonal-antibodies-or-antivirals-for-non-hospitalised-patients-with-covid-19/

12. Moreno G, Carbonell R, Martin-Loeches I, Solé-Violán J, Correig i Fraga E, Gómez J, et al. Corticosteroid treatment and mortality in mechanically ventilated COVID-19-associated acute respiratory distress syndrome (ARDS) patients: a multicentre cohort study. Ann Intensive Care. 2021 Nov 26;11:159.

13. Mozaffari E, Chandak A, Zhang Z, Liang S, Thrun M, Gottlieb RL, et al. Remdesivir Treatment in Hospitalized Patients With Coronavirus Disease 2019 (COVID-19): A Comparative Analysis of In-hospital All-cause Mortality in a Large Multicenter Observational Cohort. Clinical Infectious Diseases. 2021 Oct 1;ciab875.

14. Gupta S, Wang W, Hayek SS, Chan L, Mathews KS, Melamed ML, et al. Association Between Early Treatment With Tocilizumab and Mortality Among Critically Ill Patients With COVID-19. JAMA Internal Medicine. 2021 Jan 1;181(1):41–51.

15. Pafundi PC, Galiero R, Simeon V, Rinaldi L, Perrella A, Vetrano E, et al. Lack of effect on in-hospital mortality of drugs used during COVID-19 pandemic: Findings of the retrospective multicenter COVOCA study. PLOS ONE. 2021 Sep 14;16(9):e0256903.

16. Prats-Uribe A, Sena AG, Lai LYH, Ahmed WUR, Alghoul H, Alser O, et al. Use of repurposed and adjuvant drugs in hospital patients with covid-19: multinational network cohort study. BMJ. 2021 May 11;373:n1038.

17. Roth GA, Emmons-Bell S, Alger HM, Bradley SM, Das SR, de Lemos JA, et al. Trends in Patient Characteristics and COVID-19 In-Hospital Mortality in the United States During the COVID-19 Pandemic. JAMA Network Open. 2021 May 3;4(5):e218828.

18. Warren LR, Clarke J, Arora S, Darzi A. Improving data sharing between acute hospitals in England: an overview of health record system distribution and retrospective observational analysis of inter-hospital transitions of care. BMJ Open. 2019 Dec 1;9(12):e031637.

19. NHS Scotland. HEPMA | eHealth [Internet]. [cited 2021 Jun 8]. Available from: https://www.ehealth.scot/case-studies/hepma/

20. Scottish Government. eHealth Strategy 2014-2017 [Internet]. Edinburgh, UK: Scottish Government; 2015 Sep [cited 2020 Oct 15]. Available from: https://www.gov.scot/publications/ehealth-strategy-2014-2017/

21. NHS Digital. Electronic Prescribing and Administration (EPMA) Data in Secondary Care under the COVID-19 Direction [Internet]. NHS Digital. 2022 [cited 2022 Feb 16]. Available from: https://digital.nhs.uk/data-and-information/data-collections-and-data-

For peer review only

For peer review only - http://bmjopen.bmj.com/site/about/guidelines.xhtml

sets/data-collections/electronic-prescribing-and-administration-epma-data-in-secondary-care

22. NHS Scotland. Scotland's Health on the Web – Putting Scotland's Health on the Web [Internet]. 2022 [cited 2022 Feb 16]. Available from: https://www.scot.nhs.uk/

23. NHS England. NHS England » About us [Internet]. 2022 [cited 2022 Feb 16]. Available from: https://www.england.nhs.uk/about/

24. Information Services Division. Data Dictionary A-Z: CHI number [Internet]. ISD Scotland Data Dictionary. [cited 2020 Oct 15]. Available from: https://www.ndc.scot.nhs.uk/Dictionary-A-Z/Definitions/index.asp?ID=128&Title=CHI%20Number

25. Public Health Scotland. Population Estimates - Scottish Health and Social Care Open Data [Internet]. 2021 [cited 2021 Jun 14]. Available from: https://www.opendata.nhs.scot/dataset/population-estimates

26. University of Edinburgh. EAVE II: Early Pandemic Evaluation and Enhanced Surveillance of COVID-19 [Internet]. The University of Edinburgh. 2021 [cited 2021 Nov 23]. Available from: https://www.ed.ac.uk/usher/eave-ii

27. Mueller T, Kerr S, McTaggart S, Kurdi A, Vasileiou E, Docherty A, et al. Retrospective cohort study to evaluate medication use in patients hospitalised with COVID-19 in Scotland: protocol for a national observational study. BMJ Open. 2021 Nov 1;11(11):e054861.

28. NHS Digital. Master Person Service (MPS) [Internet]. NHS Digital. 2020 [cited 2022 Feb 16]. Available from: https://digital.nhs.uk/services/master-person-service

29. NHS Digital. Hospital Episode Statistics (HES) [Internet]. NHS Digital. 2022 [cited 2022 Feb 16]. Available from: https://digital.nhs.uk/data-and-information/data-tools-and-services/data-services/hospital-episode-statistics

30. von Elm E, Altman DG, Egger M, Pocock SJ, Gøtzsche PC, Vandenbroucke JP. Strengthening the reporting of observational studies in epidemiology (STROBE) statement: guidelines for reporting observational studies. BMJ. 2007 Oct 18;335(7624):806–8.

31. Scottish Government. The Scottish Index of Multiple Deprivation [Internet]. Statistics. 2020 [cited 2020 Oct 15]. Available from: https://www2.gov.scot/SIMD

32. DataLab. Prescribing Data: BNF Codes [Internet]. 2017 [cited 2022 Feb 16]. Available from: https://www.thedatalab.org/blog/2017/04/prescribing-data-bnf-codes/

33. UK Government. Cases in the UK | Coronavirus in the UK [Internet]. 2022 [cited 2022 Mar 4]. Available from: https://coronavirus.data.gov.uk/details/cases

For peer review only - http://bmjopen.bmj.com/site/about/guidelines.xhtml

34. UK Government. English indices of deprivation 2010 [Internet]. GOV.UK. 2011 [cited 2022 Feb 16]. Available from: https://www.gov.uk/government/statistics/english-indices-of-deprivation-2010

35. Clift AK, Coupland CAC, Keogh RH, Diaz-Ordaz K, Williamson E, Harrison EM, et al. Living risk prediction algorithm (QCOVID) for risk of hospital admission and mortality from coronavirus 19 in adults: national derivation and validation cohort study. BMJ. 2020 Oct 20;371:m3731.

36. SPICe. Timeline of Coronavirus (COVID-19) in Scotland [Internet]. SPICe Spotlight | Solas air SPICe. 2022 [cited 2022 Feb 15]. Available from: https://spice-spotlight.scot/2022/02/11/timeline-of-coronavirus-covid-19-in-scotland/

37. Wong CKH, Lau KTK, Au ICH, Xiong X, Lau EHY, Cowling BJ. Clinical Improvement, Outcomes, Antiviral Activity, and Costs Associated With Early Treatment With Remdesivir for Patients With Coronavirus Disease 2019 (COVID-19). Clinical Infectious Diseases. 2021 Jul 15;ciab631.

Figure legend

Figure 1: Prescribing of drugs of interest over time in Scotland (includes all use, regardless of treatment modality – i.e. whether drugs were used on their own or in combination)

For peer review only

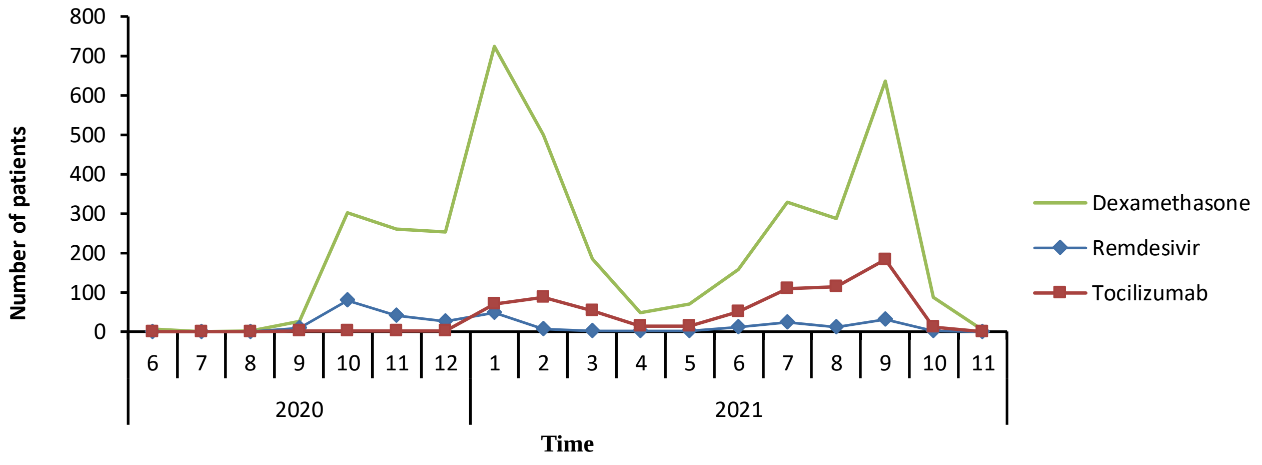

For peer review only

Supplementary material

1. Patient baseline by drug (Scotland)
2. Confirmed COVID-19 cases in Scotland, March 2020 to November 2021
3. Partial replication of analyses in England

Supplement 1: Patient baseline characteristics in Scotland by drug

Table S1: Patient baseline characteristics, monotherapy by individual drug, Scotland

n (%)	Dexamethasone n=3,094	Remdesivir n=68	Tocilizumab n=145
Socio-demographics			
Median age (IQR) [years]	66 (54 – 78)	65.5 (55 – 74)	57 (45 – 66)
Age 18 – 40 [years]	342 (11.1)	5 (7.4)	31 (21.4)
Age 41 – 70 [years]	1,475 (47.7)	39 (57.4)	87 (60.0)
Age > 70 [years]	1,277 (41.3)	24 (35.3)	27 (18.6)
Sex [male]	1,659 (53.6)	40 (58.8)	91 (62.8)
Most deprived [quintile][1]	927 (30.0)	31 (45.6)	42 (29.0)
Least deprived [quintile][1]	280 (9.0)	8 (11.8)	14 (9.7)
Region: Health Board			
Ayrshire & Arran	779 (25.2)	2 (2.9)	23 (15.9)
Dumfries & Galloway	156 (5.0)	4 (5.9)	5 (3.4)
Forth Valley	656 (21.2)	1 (1.5)	19 (13.1)
Greater Glasgow & Clyde	461 (14.9)	6 (8.8)	13 (9.0)
Lanarkshire	906 (29.3)	53 (77.9)	76 (52.4)
Lothian	136 (4.4)	2 (2.9)	9 (6.2)
Vaccination status			
Unvaccinated	2,316 (74.9)	63 (92.6)	94 (64.8)
1 dose	125 (4.0)	1 (1.5)	40 (27.6)
2 doses	653 (21.1)	4 (5.9)	11 (7.6)
Medication at baseline: number of different items[2]			
0	294 (9.5)	4 (5.9)	34 (23.5)
1 – 4	613 (19.2)	15 (22.1)	43 (29.7)
5 – 10	1,041 (33.7)	21 (30.9)	38 (26.2)
> 10	1,146 (37.0)	28 (41.2)	30 (20.7)
Medication at baseline: specific drug classes [yes][2]			
Antihypertensive drugs	1,671 (54.0)	41 (60.3)	51 (35.2)
Antithrombotic drugs	1,093 (35.3)	22 (32.3)	36 (24.8)
Statins	1,187 (38.4)	31 (45.6)	34 (23.5)
Bronchodilator	793 (25.6)	17 (25.0)	25 (17.2)
Inhaled steroid	643 (20.8)	16 (23.5)	23 (15.9)
Diabetes medication	607 (19.6)	10 (14.7)	21 (14.5)

[1] Deprivation based on the Scottish Index of Multiple Deprivation quintiles, where 1=most deprived and 5=least deprived (31)
[2] Polypharmacy and medication at baseline based on prescriptions dispensed in community pharmacy during the 6-months period directly preceding the admission, identified using BNF codes (32); includes both acute and chronic medication

For peer review only

Table S2: Patient baseline characteristics, combination therapy, Scotland

n (%)	Dexamethasone & remdesivir n=185	Dexamethasone & tocilizumab n=530	Remdesivir & tocilizumab n=10	Dexamethasone, remdesivir & tocilizumab n=31
Socio-demographics				
Median age (IQR) [years]	65 (52 – 73.5)	58 (45 – 70)	66 (60 – 74)	56 (46 – 65)
Age 18 – 40 [years]	21 (11.4)	100 (18.8)	0	5 (16.1)
Age 41 – 70 [years]	101 (54.9)	303 (57.1)	6 (60.0)	22 (71.0)
Age > 70 [years]	62 (33.7)	128 (24.1)	4 (40.0)	4 (12.9)
Sex [male]	107 (58.2)	336 (63.3)	7 (70.0)	20 (64.5)
Most deprived [quintile][1]	57 (31.0)	156 (29.4)	3 (30.0)	11 (35.5)
Least deprived [quintile][1]	18 (9.8)	58 (10.9)	0	5 (16.3)
Region: Health Board				
Ayrshire & Arran	5 (2.7)	59 (11.1)	0	0
Dumfries & Galloway	17 (9.2)	62 (11.7)	1 (10.0)	3 (9.7)
Forth Valley	22 (12.0)	60 (11.3)	0	0
GGC	43 (23.4)	72 (13.6)	4 (40.0)	15 (48.4)
Lanarkshire	85 (46.2)	224 (42.2)	4 (40.0)	5 (16.1)
Lothian	12 (6.5)	54 (10.2)	1 (10.0)	8 (25.8)
Vaccination status				
Unvaccinated	148 (80.4)	321 (60.5)	7 (70.0)	15 (48.4)
1 dose	3 (1.6)	28 (5.3)	0	4 (12.9)
2 doses	33 (17.9)	182 (34.3)	3 (30.0)	12 (38.7)
Medication at baseline: number of different items[2]				
0	15 (8.2)	97 (19.3)	2 (20.0)	1 (3.2)
1 – 4	40 (21.7)	141 (26.6)	3 (30.0)	9 (29.0)
5 – 10	60 (32.6)	162 (30.5)	3 (30.0)	14 (45.2)
> 10	69 (37.5)	131 (24.7)	2 (20.0)	7 (22.6)
Medication at baseline: specific drug classes [yes][2]				
Antihypertensive drugs	103 (56.0)	248 (46.7)	5 (50.0)	13 (41.9)
Antithrombotic drugs	57 (31.0)	129 (24.3)	4 (40.0)	7 (22.6)
Statins	74 (40.2)	173 (32.6)	4 (40.0)	8 (25.8)
Bronchodilator	57 (31.0)	85 (16.0)	2 (20.0)	5 (16.1)
Inhaled steroid	44 (23.9)	80 (15.1)	2 (20.0)	6 (19.4)
Diabetes medication	35 (19.0)	98 (18.5)	3 (30.0)	4 (12.9)

[1] Deprivation based on the Scottish Index of Multiple Deprivation quintiles, where 1=most deprived and 5=least deprived (31)

[2] Polypharmacy and medication at baseline based on prescriptions dispensed in community pharmacy during the 6-months period directly preceding the admission, identified using BNF codes (32); includes both acute and chronic medication

For peer review only

Supplement 2: confirmed COVID-19 cases in Scotland during the study period

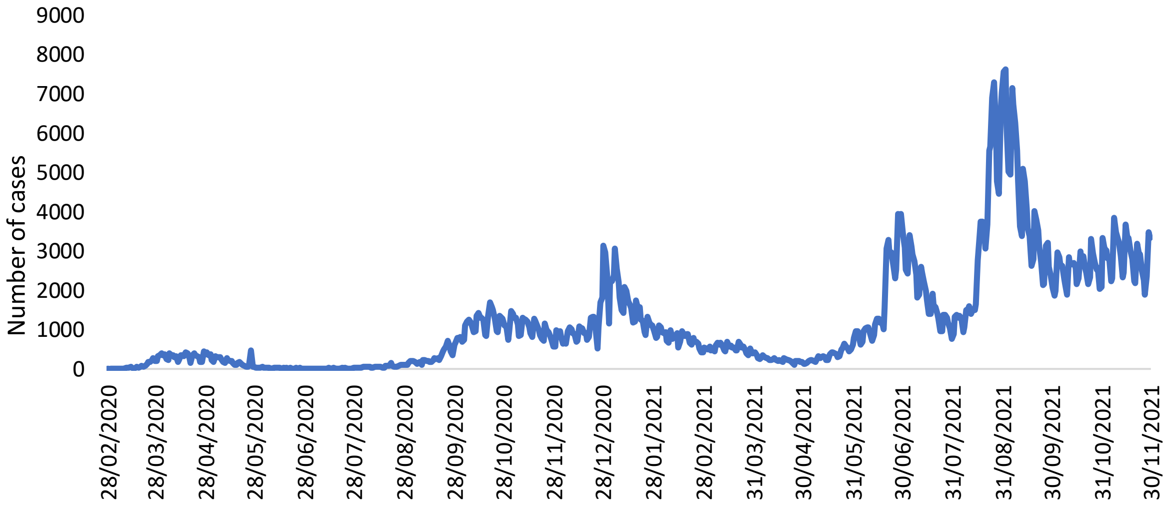

Figure S1: Confirmed COVID-19 cases in Scotland between March 2020 and November 2021 (data available online through GOV.UK (33))

Supplement 3: Partial replication of analyses in England

Context

In response to the COVID-19 pandemic, NHS Digital has established a regular collection of data from EPMA, covering up to 64 hospitals which have agreed to participate. However, Trusts do not currently use their EPMA system to record all prescribing and administration – for instance, certain specialties (e.g. ICUs) may not be recorded in the EPMA system, and the coverage and what is/is not included varies from Trust to Trust NHS Digital (21).

Patient baseline

Table S3: Patient baseline characteristics in England

n (%)	England n=7,680
Socio-demographics	
Median age (IQR) [years]	68 (55 – 81)
Age 18 – 40 [years]	671 (8.7)
Age 41 – 70 [years]	3,405 (44.3)
Age > 70 [years]	3,604 (46.8)
Sex [male]	4,384 (57.0)
Most deprived [quintile][1]	1,321 (17.2)
Least deprived [quintile][1]	1,392 (18.1)

[1] Deprivation based on the English Index of Multiple Deprivation quintiles, where 1=most deprived and 5=least deprived (34)

Medication use patterns

Table S4: Patterns of drug use in England between 1st March 2020 and 10th November 2021

n (%)	Overall[1]	Dexamethasone[2]	Remdesivir[2]	Tocilizumab[2]	More than one drug[3]
Total	7,680	7,002 (91.2)	143 (1.9)	46 (0.6)	489 (6.4)
Mar – Jun 2020	114	104 (91.2)	1 (0.9)	5 (4.4)	4 (3.5)
Jul – Sep 2021	109	93 (85.3)	5 (4.6)	0	11 (10.1)
Oct – Dec 2020	1,834	1,671 (91.1)	41 (2.2)	3 (0.2)	119 (6.5)
Jan – Mar 2021	3,705	3,499 (94.4)	55 (1.5)	16 (0.4)	135 (3.6)
Apr – June 2021	146	126 (86.3)	4 (2.7)	0	16 (11.0)
Jul – Sep 2021	1,229	1,037 (84.4)	23(1.9)	21 (1.7)	148 (12.0)
Oct – Nov 2021	543	472 (86.9)	14 (2.6)	1 (0.2)	56 (10.3)

[1] Includes all patients treated with at least one of the drugs of interest during the study period; dates of inclusion of each drug in NHS treatment guidelines: remdesivir 29 May 2020; dexamethasone 16 June 2020; tocilizumab 8 January 2021
[2] includes only patients treated exclusively with one of the drugs of interest
[3] includes patients treated with two or more of the drugs of interest, in any combination/sequence
[4] includes patients admitted to hospital up to and including 10th November, 2021

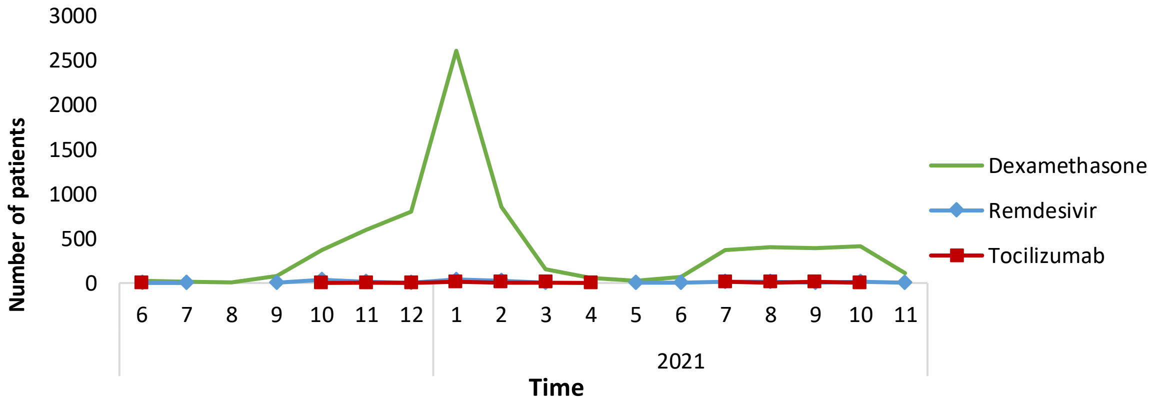

Figure S2: Prescribing of drugs over time in England

STROBE Statement—checklist of items that should be included in reports of observational studies (**Assessing medication use in COVID-19**)

	Item No.	Recommendation	Page No.
**Title and abstract**	1	(*a*) Indicate the study's design with a commonly used term in the title or the abstract	Title page
		(*b*) Provide in the abstract an informative and balanced summary of what was done and what was found	1
**Introduction**			
Background/rationale	2	Explain the scientific background and rationale for the investigation being reported	3,4
Objectives	3	State specific objectives, including any prespecified hypotheses	4
**Methods**			
Study design	4	Present key elements of study design early in the paper	4
Setting	5	Describe the setting, locations, and relevant dates, including periods of recruitment, exposure, follow-up, and data collection	4-6
Participants	6	(*a*) *Cohort study*—Give the eligibility criteria, and the sources and methods of selection of participants. Describe methods of follow-up<br>*Case-control study*—Give the eligibility criteria, and the sources and methods of case ascertainment and control selection. Give the rationale for the choice of cases and controls<br>*Cross-sectional study*—Give the eligibility criteria, and the sources and methods of selection of participants	5,6
		(*b*) *Cohort study*—For matched studies, give matching criteria and number of exposed and unexposed<br>*Case-control study*—For matched studies, give matching criteria and the number of controls per case	n/a (no matching)
Variables	7	Clearly define all outcomes, exposures, predictors, potential confounders, and effect modifiers. Give diagnostic criteria, if applicable	6
Data sources/ measurement	8*	For each variable of interest, give sources of data and details of methods of assessment (measurement). Describe comparability of assessment methods if there is more than one group	5
Bias	9	Describe any efforts to address potential sources of bias	n/a (purely descriptive study)
Study size	10	Explain how the study size was arrived at	n/a (all available patients)

Continued on next page

For peer review only - http://bmjopen.bmj.com/site/about/guidelines.xhtml

Quantitative variables	11	Explain how quantitative variables were handled in the analyses. If applicable, describe which groupings were chosen and why	6
Statistical methods	12	(*a*) Describe all statistical methods, including those used to control for confounding	6
		(*b*) Describe any methods used to examine subgroups and interactions	n/a (purely descriptive study)
		(*c*) Explain how missing data were addressed	n/a (purely descriptive study)
		(*d*) *Cohort study*—If applicable, explain how loss to follow-up was addressed<br>*Case-control study*—If applicable, explain how matching of cases and controls was addressed<br>*Cross-sectional study*—If applicable, describe analytical methods taking account of sampling strategy	n/a
		(*e*) Describe any sensitivity analyses	n/a (purely descriptive study)
**Results**			
Participants	13*	(a) Report numbers of individuals at each stage of study—eg numbers potentially eligible, examined for eligibility, confirmed eligible, included in the study, completing follow-up, and analysed	7 (all eligible patients have been included in descriptive analyses)
		(b) Give reasons for non-participation at each stage	n/a
		(c) Consider use of a flow diagram	
Descriptive data	14*	(a) Give characteristics of study participants (eg demographic, clinical, social) and information on exposures and potential confounders	8
		(b) Indicate number of participants with missing data for each variable of interest	n/a (available data on the exposure was the outcome of interest)
		(c) *Cohort study*—Summarise follow-up time (eg, average and total amount)	n/a
Outcome data	15*	*Cohort study*—Report numbers of outcome events or summary measures over time	8-10
		Case-control study—Report numbers in each exposure category, or summary measures of exposure	
		Cross-sectional study—Report numbers of outcome events or summary measures	
Main results	16	(*a*) Give unadjusted estimates and, if applicable, confounder-adjusted estimates and their precision (eg, 95% confidence interval). Make clear which confounders were adjusted for and why they were included	n/a (purely descriptive study)
		(*b*) Report category boundaries when continuous variables were categorized	n/a
		(*c*) If relevant, consider translating estimates of relative risk into absolute risk for a meaningful time period	n/a

Continued on next page

For peer review only - http://bmjopen.bmj.com/site/about/guidelines.xhtml

Other analyses	17	Report other analyses done—eg analyses of subgroups and interactions, and sensitivity analyses	n/a
**Discussion**			
Key results	18	Summarise key results with reference to study objectives	10
Limitations	19	Discuss limitations of the study, taking into account sources of potential bias or imprecision. Discuss both direction and magnitude of any potential bias	13
Interpretation	20	Give a cautious overall interpretation of results considering objectives, limitations, multiplicity of analyses, results from similar studies, and other relevant evidence	10-12
Generalisability	21	Discuss the generalisability (external validity) of the study results	12,13
**Other information**			
Funding	22	Give the source of funding and the role of the funders for the present study and, if applicable, for the original study on which the present article is based	14

*Give information separately for cases and controls in case-control studies and, if applicable, for exposed and unexposed groups in cohort and cross-sectional studies.

Note: An Explanation and Elaboration article discusses each checklist item and gives methodological background and published examples of transparent reporting. The STROBE checklist is best used in conjunction with this article (freely available on the Web sites of PLoS Medicine at http://www.plosmedicine.org/, Annals of Internal Medicine at http://www.annals.org/, and Epidemiology at http://www.epidem.com/). Information on the STROBE Initiative is available at www.strobe-statement.org.

For peer review only

BMJ Open

Assessing medication use patterns in patients hospitalised with COVID-19: a retrospective study

Journal:	*BMJ Open*
Manuscript ID	bmjopen-2022-064320.R1
Article Type:	Original research
Date Submitted by the Author:	05-Oct-2022
Complete List of Authors:	Mueller, Tanja; University of Strathclyde, Strathclyde Institute of Pharmacy and Biomedical Sciences; Public Health Scotland Glasgow Office, Clinical and Protecting Health Directorate<br>Kurdi, Amanj; University of Strathclyde, Strathclyde Institute of Pharmacy and Biomedical Sciences; Public Health Scotland Glasgow Office, Clinical and Protecting Health Directorate<br>Hall, Elliott; Public Health Scotland Glasgow Office, Clinical and Protecting Health Directorate<br>Bullard, Ian; NHS Digital<br>Wapshott, Jo; NHS Digital<br>Goodfellow, Anna; NHS Digital<br>Platt, Niketa; NHS Fife; Public Health Scotland Glasgow Office, Clinical and Protecting Health Directorate<br>Proud, Euan; Public Health Scotland Glasgow Office, Clinical and Protecting Health Directorate<br>McTaggart, Stuart; Public Health Scotland, Clinical and Protecting Health Directorate<br>Bennie, Marion; University of Strathclyde, Strathclyde Institute of Pharmacy and Biomedical Sciences; Public Health Scotland, Clinical and Protecting Health Directorate<br>Sheikh, Aziz; The University of Edinburgh Usher Institute of Population Health Sciences and Informatics; HDR UK, BREATHE Hub
**Primary Subject Heading**:	Pharmacology and therapeutics
Secondary Subject Heading:	Health informatics
Keywords:	COVID-19, THERAPEUTICS, CLINICAL PHARMACOLOGY, Health informatics < BIOTECHNOLOGY & BIOINFORMATICS

SCHOLARONE™
Manuscripts

I, the Submitting Author has the right to grant and does grant on behalf of all authors of the Work (as defined in the below author licence), an exclusive licence and/or a non-exclusive licence for contributions from authors who are: i) UK Crown employees; ii) where BMJ has agreed a CC-BY licence shall apply, and/or iii) in accordance with the terms applicable for US Federal Government officers or employees acting as part of their official duties; on a worldwide, perpetual, irrevocable, royalty-free basis to BMJ Publishing Group Ltd ("BMJ") its licensees and where the relevant Journal is co-owned by BMJ to the co-owners of the Journal, to publish the Work in this journal and any other BMJ products and to exploit all rights, as set out in our licence.

The Submitting Author accepts and understands that any supply made under these terms is made by BMJ to the Submitting Author unless you are acting as an employee on behalf of your employer or a postgraduate student of an affiliated institution which is paying any applicable article publishing charge ("APC") for Open Access articles. Where the Submitting Author wishes to make the Work available on an Open Access basis (and intends to pay the relevant APC), the terms of reuse of such Open Access shall be governed by a Creative Commons licence – details of these licences and which Creative Commons licence will apply to this Work are set out in our licence referred to above.

Other than as permitted in any relevant BMJ Author's Self Archiving Policies, I confirm this Work has not been accepted for publication elsewhere, is not being considered for publication elsewhere and does not duplicate material already published. I confirm all authors consent to publication of this Work and authorise the granting of this licence.

For peer review only

Assessing medication use patterns in patients hospitalised with COVID-19: a retrospective study

Authors: Tanja Mueller[1,2], Amanj Kurdi[1,2], Elliott Hall[2], Ian Bullard[3], Jo Wapshott[3], Anna Goodfellow[3], Niketa Platt[2,4], Euan Proud [2], Stuart McTaggart[2], Marion Bennie[1,2], Aziz Sheikh[5,6] on behalf of the EAVE II Collaboration

Affiliations: [1]Strathclyde Institute of Pharmacy and Biomedical Sciences, University of Strathclyde, Glasgow, UK; [2]Clinical and Protecting Health Directorate, Public Health Scotland, Glasgow/Edinburgh, UK; [3]NHS Digital, Leeds, UK; [4]NHS Fife, Kirkcaldy, UK; [5]Usher Institute, University of Edinburgh, Edinburgh, UK; [6]HDR UK BREATHE Hub, Edinburgh, UK

Corresponding author:

Tanja Mueller, Strathclyde Institute of Pharmacy and Biomedical Sciences, University of Strathclyde, 161 Cathedral Street, Glasgow G4 0RE, UK; email tanja.muller@strath.ac.uk

ORCID ID:

Tanja Mueller: 0000-0002-0418-4789
Amanj Kurdi: 0000-0001-5036-1988
Elliott Hall: 0000-0003-0540-0735
Stuart McTaggart: 0000-0001-6060-9019
Marion Bennie: 0000-0002-4046-629x
Aziz Sheikh: 0000-0001-7022-3056

Keywords: drug utilisation, COVID-19, in-hospital treatment, electronic prescribing

Word count: abstract: 275; main text: 3,720 (excluding tables, figures, references)

Table/figure count: 2 tables, 2 figures, online supplement comprising 1 figure and 2 tables

For peer review only

Abstract

Objective: To describe patterns of medication use – i.e. dexamethasone; remdesivir; and tocilizumab – in the management of hospitalised COVID-19 patients.

Design and setting: Retrospective observational study, using routinely collected, linked electronic data from clinical practice in Scotland. Data on drug exposure in secondary care has been obtained from the Hospital Electronic Prescribing and Medicines Administration System.

Participants: Patients being treated with the drugs of interest and hospitalised for COVID-19 between 1st March 2020 and 10th November 2021.

Outcomes: Identification of patients subject to the treatments of interest; summary of patients' baseline characteristics; description of medication use patterns and treatment episodes. Analyses were descriptive in nature.

Results: Overall, 4063 patients matching the inclusion criteria were identified in Scotland, with a median (IQR) age of 64 years (52 – 76). Among all patients, 81.4% (n=3307) and 17.8% (n=725) were treated with one or two medicines, respectively; dexamethasone monotherapy accounted for the majority (n=3094, 76.2%) followed by dexamethasone in combination with tocilizumab (n=530, 13.0%). Treatment patterns were variable over time but roughly followed the waves of COVID-19 infections; however, the different drugs were used to varying degrees during the study period.

The median (IQR) treatment duration differed by medicine: dexamethasone 5 days (2 – 9); remdesivir 5 days (2 – 5); and tocilizumab 1 day (1 – 1). The overall median (IQR) length of hospital stay among all patients included in the study cohort was 9 days (5 – 17); 24.7% of patients died in hospital.

Conclusion: The use of adjuvant medicines in hospitalised COVID-19 patients appears in line with evolving evidence and changing treatment guidelines. In-hospital electronic prescribing systems are a valuable source of information, providing detailed patient-level data on in-hospital drug use.

For peer review only - http://bmjopen.bmj.com/site/about/guidelines.xhtml

For peer review only

ARTICLE SUMMARY

Strengths and limitations of this study:

- This study used patient-level data collected as part of routine care.
- Available data enabled an in-depth description of the use of repurposed drugs in patients hospitalised with COVID-19 in Scotland.
- Due to the observational nature of this study and limitations on the data, analyses of clinical safety or effectiveness of the drugs of interest were not feasible.

For peer review only

INTRODUCTION

Similar to other countries globally, COVID-19 had a considerable impact on population health in the UK; in Scotland alone, approximately 44,300 hospital admissions and 12,500 deaths have been attributed to the disease between March 2020 and December 2021 (1). Even though vaccines were rolled-out at speed, the search for effective treatments is ongoing due to limits on vaccine effectiveness (2), immune escape particularly among Variants of Concern (VoC) (3), and waning immunity following both infection and vaccination (4). While a range of diverse options were discussed early in the pandemic – spanning from antibiotics to convalescent plasma – only a small number of medicines were found to be effective in clinical trials (5), and subsequently approved for use by regulators. Medicines recommended for use in patients hospitalised with COVID-19 in the UK at the time of study conduct comprised remdesivir, an antiviral drug (introduced in guidelines 29th May 2020); steroids, most prominently dexamethasone (16th June 2020); and monoclonal antibodies including tocilizumab (8th January 2021) (6–8). Guidelines have however undergone frequent changes over the last two years (9). Dexamethasone is now routinely given to patients with low oxygen saturation, primarily because it is an effective and generally well tolerated anti-inflammatory drug, with extensive experience in usage. Remdesivir is recommended in patients receiving low-flow additional oxygen, depending on renal and liver function, to be given within the first few days of infection as it is deemed ineffective at later stages; tocilizumab is recommended in patients receiving respiratory support (10). While usually given separately, consecutive (e.g., dexamethasone together with tocilizumab) or subsequent treatment with multiple drugs can be considered based on clinical condition (10,11). Most recently, a number of antivirals and monoclonal antibodies have also been approved for use in the community, including, e.g., molnupiravir and sotrovimab (12).

There is a need for real-world studies assessing the uptake, effectiveness, and safety of these drugs to complement the findings from rapidly undertaken efficacy trials. Nevertheless, thus far, evidence from clinical practice is limited; in particular, there are very few publications offering detailed descriptions of in-hospital drug use in Scotland. While a range of studies have investigated the use of repurposed and adjuvant drugs in COVID-19

For peer review only - http://bmjopen.bmj.com/site/about/guidelines.xhtml

patients, the majority of publications have focused on the effect of specific drugs on disease outcomes rather than the drug use itself (e.g., (13–15)). Although there are exceptions (16), studies rarely provide in-depth information on treatment patterns (including temporal and geographical variation); details on duration of treatment are even less common, possibly due to the unavailability of in-depth, patient-level information (17–19).

In the UK, the drive towards digitisation in health care has resulted in the implementation of electronic prescribing; initially in primary care, but subsequently also in specialist settings (e.g., oncology) and secondary care. In Scotland, the roll-out of the Hospital Electronic Prescribing and Medicines Administration (HEPMA) system was initiated in 2014; HEPMA is now available in hospitals across six of the 14 regional organisations (Health Boards) within NHS Scotland tasked with planning and delivering services to their local populations.

The aim of this study was to investigate the use of in-hospital medication for the treatment of COVID-19, namely dexamethasone, remdesivir and tocilizumab. Our specific objectives were: to describe medicines use over time and across geographical areas; to summarise patients' baseline characteristics; and to describe treatment patterns as well as hospital admission episodes, including their outcomes.

METHODS

Study design and setting

The study was designed as a retrospective cohort study using routinely collected linked health care data, covering the time period from 1st March 2020 to 10th November 2021 based on the timeline of events (World Health Organization interim guidance/declaration of the pandemic) and data availability. Health care throughout the UK is provided via the tax-funded National Health Services (NHS), with services being offered primarily without payment at the point of care (20).

The study setting was Scotland, where every resident is allocated a unique patient identifier (the Community Health Index (CHI) number) the use of which is mandatory throughout the health and care system (21), thereby enabling deterministic linkage of electronic patient records. Since implementation of technical solutions takes place at Health Board level and

For peer review only - http://bmjopen.bmj.com/site/about/guidelines.xhtml

regional time frames depend on local requirements and circumstances, at the time of study conduct, HEPMA had been implemented fully or partially in six Health Boards: NHS Ayrshire & Arran; NHS Dumfries & Galloway; NHS Forth Valley; NHS Greater Glasgow & Clyde (GGC); NHS Lanarkshire; and NHS Lothian, covering an estimated 3.6M people or approximately 65% of the Scottish population (5.4M) (22). However, as the HEPMA implementation started at different points in time in the various Health Boards and roll-out across hospital sites was incremental, data coverage was incomplete for two Health Boards: NHS GGC, where implementation started in late 2020, i.e. after the start of the study period; and NHS Lothian, where very few hospitals were initially included. In addition, coverage within individual hospitals differed due to the use of existing systems instead of (or in addition to) HEPMA in certain areas, e.g. in intensive care units (ICUs).

Data sources

Briefly, HEPMA was used to extract any prescriptions of the medications of interest. Using CHI numbers, HEPMA was subsequently linked to the Scottish Morbidity Records inpatient dataset (SMR01) and the Electronic Communication of Surveillance Scotland (ECOSS) data to identify the study cohort and ensure that the medications of interest were administered during a COVID-19 related hospital admission episode. A number of other datasets (including, but not limited to, primary care prescriptions and death records) were also available through the Early Pandemic Evaluation and Enhanced Surveillance of COVID-19 (EAVE II) surveillance platform (23). Further details can be found in the protocol (24).

Study population

With drug use being the specific focus of the study, the main study cohort comprised patients who had received at least one of the medications of interest (i.e., dexamethasone, remdesivir, tocilizumab) while being admitted to hospital in the aforementioned Scottish Health Boards between 1st March 2020 and 10th November 2021 for suspected or confirmed COVID-19. Hospitalisations for COVID-19 were defined as admissions within 28 days of a positive reverse transcriptase polymerase chain reaction (RT-PCR) test and/or with an International Classification of Diseases, 10th edition (ICD-10) code for COVID-19 (U07.1, U07.2) recorded on a patient's hospital admission file as the primary or secondary diagnosis.

For peer review only - http://bmjopen.bmj.com/site/about/guidelines.xhtml

Drug use, covariates, and statistical analyses

The primary outcome was observed treatment patterns among patients with any of the medications of interest over the duration of the study period, including both monotherapy as well as treatment with any combinations thereof. In addition, secondary outcomes included summaries of exposure in terms of administered dose and duration of treatment; as well as length of hospital stay and in-hospital mortality.

Patient characteristics, including socio-demographic factors (25), COVID-19 vaccination status, disease-related aspects, comorbidities (26), and concomitant medication (27), were summarised at baseline. Detailed definitions, descriptions, and relevant data sources can be found in the protocol (24).

All analyses were descriptive in nature; results were expressed as counts/frequencies for categorical variables, and median/inter-quartile range (IQR) for continuous variables. Patients' index dates – i.e., the date of the first recorded prescription for any of the drugs of interest for each patient – were used to classify treatment over time. Analyses were conducted using R/R Studio V.3.6.1, and Stata 15.

Patient and public involvement

The EAVE II Public Advisory group are a diverse group of PPI contributors who meet monthly to incorporate the views of patients and the public into research using the EAVE II dataset. This includes shaping of research via the EAVE II Steering Group, which is attended by two lay leads. A lay summary, co-written by our PPI contributors, will be shared via the outputs section of the EAVE II website, hosted by the University of Edinburgh (23).

Ethical approval

Ethical and information governance approvals have been obtained by the National Research Ethics Service Committee (REC), South East Scotland 02 (REC number: 12/SS/0102) and the Public Benefit and Privacy Panel for Health and Social Care (reference number: 1920-0279), respectively. Study data were anonymised prior to being made available for analyses; and held within a secure environment with restrictions on access.

For peer review only - http://bmjopen.bmj.com/site/about/guidelines.xhtml

Reporting of the study follows the STROBE (Strengthening the Reporting of Observational Studies in Epidemiology) guidelines (28); a STROBE checklist can be found in the supplementary material.

RESULTS

At the study locations in Scotland, 4063 patients admitted to hospital with a suspected or confirmed diagnosis of COVID-19 received at least one of the drugs of interest during the study period. Of these, 3745 patients (92.2%) had COVID-19 recorded as their primary diagnosis for hospital admission; 109 patients (2.7%) did not have COVID-19 as a diagnosis on their admission records and were identified based on a positive RT-PCR test alone. The vast majority (n=3913, 96.8%) of admissions were emergency admissions, with the majority admitted via the Acute Assessment Unit significant care facility (n=2157/4063, 53.1%).

The largest group of patients was treated in NHS Lanarkshire (n=1353, 33.3%), followed by NHS Ayrshire & Arran (n=868, 21.4%); NHS Forth Valley (n=758, 18.7%); and NHS GGC (n=614, 15.1%). The remaining two Health Boards – NHS Dumfries & Galloway and NHS Lothian – accounted for relatively small patient populations (n=248 (6.1%) and n=222 (5.5%), respectively).

Patient baseline characteristics

The median age of all patients at the time of hospital admission was 64 years (IQR 52 – 76), with 37.6% of patients over 70 years of age. Just over half of the patients (55.6%) were male. While approximately half of all patients (n=2159, 53.1%) had a Charlson score of 0 indicating no comorbidities requiring hospitalisation during the five-year period prior to their COVID-19 admission the majority (n=3616, 89.0%) had received at least one prescription drug in primary care in the preceding six months. The most commonly prescribed medication included antihypertensive drugs, statins, and antithrombotic drugs; polypharmacy – i.e. being prescribed five or more different medications simultaneously– was observed among 2752 patients (67.7%). See table 1 for details, and tables S1 and S2 in the supplementary material for a breakdown by individual drug.

For peer review only - http://bmjopen.bmj.com/site/about/guidelines.xhtml

For peer review only

Table 1: Patient baseline characteristics in Scotland

n (%)	Scotland n=4,063
Socio-demographics	
Median age (IQR) [years]	64 (52 – 76)
Age 18 – 40 [years]	504 (12.4)
Age 41 – 70 [years]	2,033 (50.0)
Age > 70 [years]	1,526 (37.6)
Sex [male]	2,260 (55.6)
Most deprived [quintile][1]	1,227 (30.2)
Least deprived [quintile][1]	383 (9.4)
Vaccination status	
Unvaccinated	2,964 (73.0)
1 dose	172 (4.2)
2 doses	927 (22.8)
Charlson score[2]	
0	2,159 (53.1)
1 – 2	1,339 (33.0)
3 – 4	383 (9.4)
> 4	182 (4.5)
Medication at baseline: number of different items[3]	
0	447 (11.0)
1 – 4	864 (21.3)
5 – 10	1,339 (33.0)
> 10	1,413 (34.8)
Medication at baseline: specific drug classes [yes][3]	
Antihypertensive drugs	2,141 (52.7%)
Antithrombotic drugs	1,348 (33.2%)
Statins	1,511 (37.2%)
Bronchodilator	984 (24.2%)
Inhaled steroid	814 (20.0%)
Diabetes medication	778 (19.2%)

[1] Deprivation based on the Scottish Index of Multiple Deprivation quintiles, where 1=most deprived and 5=least deprived (25)

[2] Charlson score at baseline based on diagnoses from hospital discharge records during the 5-year period directly preceding the admission, identified using ICD-10 codes (26)

[3] Polypharmacy and medication at baseline based on prescriptions dispensed in community pharmacy during the 6-months period directly preceding the admission, identified using BNF codes (27); includes both acute and chronic medication

Medication use patterns

Treatment with dexamethasone, remdesivir, and tocilizumab in patients with COVID-19 was variable over time. Generally speaking, prescribing patterns roughly followed the waves of COVID-19 infections; however, the different medicines were used to varying degrees and at diverging points in time during the study period. While dexamethasone use was observed throughout this period, remdesivir was primarily prescribed in 2020, whereas routine tocilizumab use started in early 2021; see also figure 1, and figure S1 in the supplementary

For peer review only - http://bmjopen.bmj.com/site/about/guidelines.xhtml

material for a representation of proportional changes in drug use over time. For further context, figure 2 depicts COVID-19 hospitalisations in Scotland during the study period (29).

[figure 1] [figure 2]

Furthermore, although the majority of patients were treated with one drug only (n=3307, 81.4%), some patients received two (n=725, 17.8%) or all three (n=31, 0.8%). Most of the patients treated with more than one drug received dexamethasone and tocilizumab in combination (n=530/756, 70.1%), followed by dexamethasone and remdesivir (n=185, 24.5%). While the vast majority of the patients with multiple drugs (n=748/756, 98.9%) received those during the same hospital admission episode, overall, the timing/sequencing of the drugs given was very diverse, with no clear patterns emerging among the patients who received two or more of them. For details, see table 2.

Table 2: Patterns of drug use in Scotland between 1st March 2020 and 10th November 2021

n (%)	Overall[1]	Dexamethasone[2]	Remdesivir[2]	Tocilizumab[2]	More than one drug[3]
Scotland					
Total	4,063	3,094 (76.2)	68 (1.7)	145 (3.6)	756 (18.5)
Mar – Jun 2020	6	6 (100)	0	0	0
Jul – Sep 2020	31	21 (67.7)	3 (9.7)	0	7 (22.6)
Oct – Dec 2020	860	711 (82.7)	45 (5.2)	0	104 (12.1)
Jan – Mar 2021	1,461	1,203 (82.3)	15 (1.0)	55 (3.8)	188 (12.9)
Apr – June 2021	290	199 (68.8)	1 (0.3)	16 (5.5)	74 (25.5)
Jul – Sep 2021	1,324	874 (66.0)	4 (0.3)	73 (5.5)	373 (28.2)
Oct – Nov 2021[4]	91	80 (87.9)	0	1 (1.1)	10 (11.0)

[1] Includes all patients treated with at least one of the drugs of interest during the study period; dates of inclusion of each drug in NHS treatment guidelines: remdesivir 29 May 2020; dexamethasone 16 June 2020; tocilizumab 8 January 2021 (6–8)
[2] includes only patients treated exclusively with one of the drugs of interest
[3] includes patients treated with two or more of the drugs of interest, in any combination/sequence
[4] includes patients admitted to hospital up to and including 10th November, 2021

Treatment episodes

Patients usually started treatment very soon after admission, with a median time between hospital admission and treatment initiation of 1 day (IQR 1 – 2) across the entire Scottish cohort; there was very little difference with regards to treatment initiation between the individual drugs or any of the combinations thereof. The median treatment duration overall was 4 days (IQR 1 – 8) but this differed by drug and whether patients were treated with one drug only, or a combination of drugs: individually, dexamethasone and remdesivir were

For peer review only - http://bmjopen.bmj.com/site/about/guidelines.xhtml

given for a median of 5 days (IQR 2 – 8 and 3 – 5, respectively); in contrast, among patients treated with dexamethasone and one other drug (either remdesivir or tocilizumab), median duration of dexamethasone treatment was 7 days (IQR 4 – 9). Tocilizumab was given as a single dose only (median 1 day, IQR 1 – 1) regardless of treatment modality.

While standard dosing regimens were in place for both dexamethasone and remdesivir, tocilizumab is dosed based on patient weight, which was reflected in the observed first/daily and overall doses given to patients. For dexamethasone, the median dose was 6mg once a day (IQR 6 – 6), with a median total dose of 30mg (IQR 12 – 48); however, the median total dose was lower in patients treated exclusively with dexamethasone (24mg, IQR 12 – 48) in line with the shorter duration of treatment in comparison to patients treated with a combination of drugs. For remdesivir, the starting dose was 200mg at day one followed by 100mg once daily on subsequent days for the vast majority of patients, with an overall median total dose of 600mg (IQR 300 – 600). The median dose of tocilizumab given to patients in Scotland was 800mg (IQR 600 – 800).

The overall median length of hospital stay of all patients included in the study cohort, regardless of whether patients were discharged or died, was 9 days (IQR 5 – 17), with minor differences between patients being treated with dexamethasone (median 8 days, IQR 5 – 17); remdesivir (median 11 days, IQR 8 – 18); or tocilizumab (median 13 days, IQR 8 – 24) as a single agent. The majority of patients (2807, 69.1%) were discharged to a private residence, whereas 1002 patients (24.7%) died in hospital.

DISCUSSION

Using routinely collected data, our findings provide a picture of clinical practice during the study period in Scotland and demonstrate differences in the use of specific drugs for the treatment of COVID-19, as well as changes in their use over time. Of the three drugs of interest, dexamethasone was most widely used in hospitalised COVID-19 patients in Scotland; while 76.2% of all treated patients received this steroid, only 1.7% and 3.6% of patients were treated with remdesivir and tocilizumab, respectively. Furthermore, 18.5% of patients received varying combinations of these three drugs. Although study designs and analytical methods differed widely, use of dexamethasone was also found to be high in

For peer review only - http://bmjopen.bmj.com/site/about/guidelines.xhtml

other studies: an evaluation of the adoption of corticosteroid treatment in the UK following the RECOVERY trial found that 75.2% of patients on supplementary oxygen between June 2020 and April 2021 received corticosteroids (30); an international, multicentre study reported that 68.5% of patients admitted to ICU were treated with steroids (31); and among patients hospitalised in Pakistan, the rate was 93.9% for steroids overall, and 91.2% for dexamethasone specifically – although these included patients who received combinations of steroids and other drugs (32). Conversely, treatment with dexamethasone was considerably lower among hospitalised patients who received treatment for COVID-19 in a study conducted in the US, with only 35.4%; nevertheless, in this study, another 36.6% of patients received various drug combinations that included dexamethasone (33). In contrast, use of remdesivir was found to be much higher in other countries than in Scotland, particularly in Pakistan with 45.0% of hospitalised patients (32), and the US with 11.5% of treated patients with remdesivir alone and another 26.0% of patients with various combinations containing remdesivir (33).

While many underlying reasons may have contributed to these differences in treatment, two conceivable explanations relate to the use of clinical guidelines; and the general availability of drugs. For instance, in the UK, treatment guidelines – although not strictly speaking mandatory – will usually be followed, which may have contributed to the low use of remdesivir where restrictions have been put in place for its use based on time since symptom onset and renal and liver function (34); in contrast, some shortages in the supply of dexamethasone might have impacted its use in the US (16). Of note: drug combinations reported in other studies comprised a much wider range than in our study, and included, for example, azithromycin; hydroxychloroquine; and/or ivermectin – none of which have been authorised for use in COVID-19 in the UK.

Dexamethasone was used throughout the study period, with two prominent peaks: first in January 2021, and then again in September 2021 – in line with COVID-19 waves in the UK. In contrast, remdesivir was prescribed early on (it was introduced in guidelines in late May 2020), but its use decreased soon after, being replaced by tocilizumab from January 2021 onwards. Observed changes in treatment pattern over time are, however, in line with evolving knowledge and, consequently, changes in treatment guidelines (9), and have also

For peer review only - http://bmjopen.bmj.com/site/about/guidelines.xhtml

been reported in other studies: an English study for example reported a decrease in the use of remdesivir, and an increase in the use of tocilizumab, during their study period (35). Interestingly, the number of patients receiving more than one of the studied drugs – most prominently, dexamethasone and tocilizumab – increased over time; anecdotally, the addition of dexamethasone to both tocilizumab and remdesivir treatment has been described in other observational studies, mostly from the US (e.g. (15,33)). Since there was wide variety in both the combination and sequencing of drugs and treatment decisions might have been influenced by a number of factors, including disease severity and other, unknown patient-related aspects, interpretation of these findings is very difficult without further information and would, at this point, be merely speculative.

Duration of treatment was mostly in line with guidelines, with a median of five days for remdesivir and a single dose of tocilizumab; similar findings regarding remdesivir have been described in a study conducted in Hong Kong (36). However, duration of dexamethasone treatment appeared shorter than recommended, with a median of five instead of 10 days – albeit with a rather wide confidence interval of two to nine days. While differences in median duration of treatment depended on whether patients were ventilated in a study from the US (16), reasons underlying our findings are unclear, but could potentially be related to patients being moved to a different ward with no HEPMA coverage (e.g., ICU due to deterioration of condition); or being discharged. Alternatively, patients might indeed have stopped treatment with dexamethasone earlier than recommended. Unfortunately, reasons for neither initiating nor discontinuing treatment are recorded on HEPMA.

In Scotland, the majority of patients being treated with any of the three drugs of interest while admitted to hospital for COVID-19, as captured by the available in-hospital prescribing data, were elderly, with a median age of 64 years; furthermore, prescribing data from community care indicated that patients had a range of existing comorbidities, including diabetes, asthma/COPD, hypertension, and possibly other cardiovascular diseases. This is in line with expectations based on existing evidence, as disease severity – and, consequently, hospitalisation rates and/or disease outcomes – were shown to be associated with patient age and the presence of certain conditions (37). Since drug use is closely linked with age (generally speaking, the older a patient, the more medications they will be on) the high level

For peer review only - http://bmjopen.bmj.com/site/about/guidelines.xhtml

of polypharmacy observed among the study cohort was not entirely unexpected, even though the extent of excessive polypharmacy – i.e. 10 or more different drugs – was somewhat surprising; this might, however, have been influenced by the method of analysis, as the number of drugs prescribed to patients reflects both chronic and acute medication and could have been inflated by not accounting for treatment changes (e.g. patients switching from one anti-hypertensive drug to another during the assessed time period).

As an aside, while the majority of patients captured in this study (73.0%) were unvaccinated, this might primarily be ascribable to the timing of the study, since a large part of the cohort stems from before the vaccination campaign was rolled-out across Scotland. Vaccinations, initially among NHS and care home staff and care home residents, commenced on 8th December 2020, and were subsequently offered in stages; first to clinically vulnerable populations and the elderly, before being available to all adult residents and, subsequently, children aged 5 years and above (38).

When interpreting results, two further relevant features of prescribing practices need to be taken into account. First, dexamethasone is not the only steroid to be used in patients with COVID-19 (prednisolone is, e.g., used in pregnant patients) and, due to intermittent drug shortages, tocilizumab has on occasion been replaced by sarilumab (amongst others). As these alternatives were not included in the analyses, total drug use may have been underestimated within the cohort. Second, the three drugs of interest are given to different groups of patients, at different points in time on their disease journey. Accordingly, direct comparison between the use of the different drugs, the patients receiving them, and – most importantly – observed patient outcomes, may not be particularly meaningful without considering the complexity of cases and possible variations in treatment based on patient, prescriber, and/or wider contextual aspects.

Study limitations

Although the use of HEPMA offers unprecedented opportunities to conduct drug utilisation research within a secondary care setting, this study also has a number of limitations, which need to be considered when interpreting findings. Most importantly, data coverage was variable between as well as within Health Boards. Some of the major hospitals in NHS

For peer review only - http://bmjopen.bmj.com/site/about/guidelines.xhtml

Lothian were, for instance, not captured, and data from ICUs were missing as these do not routinely use HEPMA. This may have affected the accuracy of findings; for instance, the number of patients treated with the medicines of interest – in particular, tocilizumab – might have been underestimated due to its use in ICUs. In addition, there are implications on the comparability of findings. Direct comparison of treatment patterns and patient characteristics across Scottish Health Boards, for example, is inadvisable at this point due to the incremental roll-out of the system. Furthermore, although there were clear eligibility and prescribing criteria in place for all three drugs included in this study, we were not able to assess eligibility for treatment; this should, however, not have impacted our findings since the study aim was to describe patterns of use in clinical practice. Finally, caution is advised when interpreting patient characteristics. This study presents a snapshot of hospitalised COVID-19 patients, describing only a sample of patients admitted to hospital who started treatment with any of the drugs of interest in areas where HEPMA has been implemented. Consequently, these characteristics should not be considered representative of all hospitalised COVID-19 patients in Scotland.

CONCLUSION

In conclusion, the use of adjuvant medicines in hospitalised COVID-19 patients in Scotland appears in line with evolving evidence and changing treatment guidelines; nevertheless, findings with regards to the use of multiple drugs and the duration of dexamethasone treatment require further investigation.

Author contributions: TM, AK, SM, MB conceptualised and designed the study. TM, AK, EH curated the data and performed the analyses; IB, JW, AG performed additional analyses. NP, EP, SM, MB, AS contributed to data interpretation. MB and AS had the clinical and academic oversight of the project. All authors have contributed to drafting the paper or revising it critically and have approved of the final version.

EAVE II Collaboration: Colin R Simpson, School of Health, Victoria University of Wellington, Wellington, New Zealand and Usher Institute, University of Edinburgh, Edinburgh, UK; Holly Tibble, Usher Institute, University of Edinburgh, Edinburgh, UK; Chris Robertson, Public

For peer review only - http://bmjopen.bmj.com/site/about/guidelines.xhtml

Health Scotland, Glasgow, UK and Department of Mathematics and Statistics, University of Strathclyde, UK; critically reviewed the study proposal and the draft manuscript.

Acknowledgements: We thank Wendy Inglis-Humphrey and Vicky Hammersley for their support with project management and administration. This work is only possible because of the wealth of information collected by the NHS as part of routine care.

Funding information: This study is part of the EAVE II project. EAVE II is funded by the Medical Research Council [MC_PC_19075] with the support of BREATHE – The Health Data Research Hub for Respiratory Health [MC_PC_19004], which in turn is funded through the UK Research and Innovation Industrial Strategy Challenge Fund and delivered through Health Data Research UK. Additional support has been provided through the Scottish Government DG Health and Social Care and the Chief Scientist's Office of the Scottish Government (no grant/award number).

Competing interests: AS reports grants from NIHR, MRC, and HDR-UK; and MB, AK, and TM report grants from HDR-UK during the conduct of this study. All other authors report no potential conflicts of interest.

Data availability statement: NHS data are confidential and only available upon request subject to approval by a Caldicott Guardian/the Public Benefit and Privacy Panel for Health and Social Care in Scotland, and the Data Access Request Service in England.

Patient consent for publication: Not required.

Ethics approval statement: Ethical and information governance approvals have been obtained by the National Research Ethics Service Committee (REC), South East Scotland 02 (REC number: 12/SS/0102) and the Public Benefit and Privacy Panel for Health and Social Care (reference number: 1920-0279), respectively. Study data were anonymised prior to being made available for analyses; and held within a secure environment with restrictions on access. The need for consent was waived by the ethics committee.

For peer review only

For peer review only - http://bmjopen.bmj.com/site/about/guidelines.xhtml

References

1. Scottish Government. COVID-19 | Data [Internet]. 2022 [cited 2022 Sep 16]. Available from: https://data.gov.scot/coronavirus-covid-19/data.html
2. Institute for Health Metrics and Evaluation. COVID-19 vaccine efficacy summary [Internet]. Institute for Health Metrics and Evaluation. 2022 [cited 2022 Feb 25]. Available from: https://www.healthdata.org/covid/covid-19-vaccine-efficacy-summary
3. Hu J, Peng P, Cao X, Wu K, Chen J, Wang K, et al. Increased immune escape of the new SARS-CoV-2 variant of concern Omicron. Cell Mol Immunol. 2022 Feb;19(2):293–5.
4. Katikireddi SV, Cerqueira-Silva T, Vasileiou E, Robertson C, Amele S, Pan J, et al. Two-dose ChAdOx1 nCoV-19 vaccine protection against COVID-19 hospital admissions and deaths over time: a retrospective, population-based cohort study in Scotland and Brazil. The Lancet. 2022 Jan 1;399(10319):25–35.
5. Nuffield Department of Population Health. RECOVERY - Randomised Evaluation of COVID-19 Therapy [Internet]. 2022 [cited 2022 Feb 15]. Available from: https://www.recoverytrial.net/
6. Scottish Government. COVID-19 therapeutic alert: Dexamethasone in the treatment of COVID-19: implementation and management of supply for treatment in hospitals [Internet]. [cited 2021 Aug 6] p. 5. Available from: https://www.sehd.scot.nhs.uk/publications/DC20200616COVID-19Dexamethasone.pdf
7. NHS England. Coronavirus » Interim Clinical Commissioning Policy: Remdesivir for patients hospitalised with COVID-19 (adults and children 12 years and older) [Internet]. [cited 2021 Mar 23]. Available from: https://www.england.nhs.uk/coronavirus/publication/interim-clinical-commissioning-policy-remdesivir-for-patients-hospitalised-with-covid-19-adults-and-children-12-years-and-older/
8. NHS England. Coronavirus » Interim Clinical Commissioning Policy: Tocilizumab for hospitalised patients with COVID-19 pneumonia (adults) [Internet]. [cited 2021 Mar 23]. Available from: https://www.england.nhs.uk/coronavirus/publication/interim-clinical-commissioning-policy-tocilizumab-for-hospitalised-patients-patients-with-covid-19-pneumonia-adults/
9. MHRA. Central Alerting system: Coronavirus COVID-19) Alerts and Registration [Internet]. 2022. Available from: https://www.cas.mhra.gov.uk/Help/CoronavirusAlerts.aspx
10. NHS England. Clinical pathway: Therapies for patients hospitalised due to COVID-19 [Internet]. 2022 [cited 2022 Sep 29]. Available from: https://www.england.nhs.uk/coronavirus/wp-content/uploads/sites/52/2022/05/C1643-ii-clinical-pathway-therapies-for-patients-hospitalised-due-to-covid-19-guide-v3-may-2022.pdf

For peer review only - http://bmjopen.bmj.com/site/about/guidelines.xhtml

11. NHS England. Interim Clinical Commissioning Policy: IL-6 inhibitors (tocilizumab or sarilumab) for hospitalised patients with COVID-19 (adults) [Internet]. 2021 [cited 2022 Sep 29]. Available from: https://www.cas.mhra.gov.uk/ViewandAcknowledgment/ViewAlert.aspx?AlertID=103173

12. Department of Health & Social Care. Coronavirus » Interim clinical commissioning policy: neutralising monoclonal antibodies or antivirals for non-hospitalised patients with COVID-19 [Internet]. 2022 [cited 2022 Jun 7]. Available from: https://www.england.nhs.uk/coronavirus/publication/interim-clinical-commissioning-policy-neutralising-monoclonal-antibodies-or-antivirals-for-non-hospitalised-patients-with-covid-19/

13. Moreno G, Carbonell R, Martin-Loeches I, Solé-Violán J, Correig i Fraga E, Gómez J, et al. Corticosteroid treatment and mortality in mechanically ventilated COVID-19-associated acute respiratory distress syndrome (ARDS) patients: a multicentre cohort study. Ann Intensive Care. 2021 Nov 26;11:159.

14. Mozaffari E, Chandak A, Zhang Z, Liang S, Thrun M, Gottlieb RL, et al. Remdesivir Treatment in Hospitalized Patients With Coronavirus Disease 2019 (COVID-19): A Comparative Analysis of In-hospital All-cause Mortality in a Large Multicenter Observational Cohort. Clinical Infectious Diseases. 2021 Oct 1;ciab875.

15. Gupta S, Wang W, Hayek SS, Chan L, Mathews KS, Melamed ML, et al. Association Between Early Treatment With Tocilizumab and Mortality Among Critically Ill Patients With COVID-19. JAMA Internal Medicine. 2021 Jan 1;181(1):41–51.

16. Mehta HB, An H, Andersen KM, Mansour O, Madhira V, Rashidi ES, et al. Use of Hydroxychloroquine, Remdesivir, and Dexamethasone Among Adults Hospitalized With COVID-19 in the United States. Ann Intern Med. 2021 Oct 19;174(10):1395–403.

17. Pafundi PC, Galiero R, Simeon V, Rinaldi L, Perrella A, Vetrano E, et al. Lack of effect on in-hospital mortality of drugs used during COVID-19 pandemic: Findings of the retrospective multicenter COVOCA study. PLOS ONE. 2021 Sep 14;16(9):e0256903.

18. Prats-Uribe A, Sena AG, Lai LYH, Ahmed WUR, Alghoul H, Alser O, et al. Use of repurposed and adjuvant drugs in hospital patients with covid-19: multinational network cohort study. BMJ. 2021 May 11;373:n1038.

19. Roth GA, Emmons-Bell S, Alger HM, Bradley SM, Das SR, de Lemos JA, et al. Trends in Patient Characteristics and COVID-19 In-Hospital Mortality in the United States During the COVID-19 Pandemic. JAMA Network Open. 2021 May 3;4(5):e218828.

20. NHS Scotland. Scotland's Health on the Web – Putting Scotland's Health on the Web [Internet]. 2022 [cited 2022 Feb 16]. Available from: https://www.scot.nhs.uk/

21. Information Services Division. Data Dictionary A-Z: CHI number [Internet]. ISD Scotland Data Dictionary. [cited 2020 Oct 15]. Available from:

For peer review only

For peer review only - http://bmjopen.bmj.com/site/about/guidelines.xhtml

https://www.ndc.scot.nhs.uk/Dictionary-A-Z/Definitions/index.asp?ID=128&Title=CHI%20Number

22. Public Health Scotland. Population Estimates - Scottish Health and Social Care Open Data [Internet]. 2021 [cited 2021 Jun 14]. Available from: https://www.opendata.nhs.scot/dataset/population-estimates

23. University of Edinburgh. EAVE II: Early Pandemic Evaluation and Enhanced Surveillance of COVID-19 [Internet]. The University of Edinburgh. 2021 [cited 2021 Nov 23]. Available from: https://www.ed.ac.uk/usher/eave-ii

24. Mueller T, Kerr S, McTaggart S, Kurdi A, Vasileiou E, Docherty A, et al. Retrospective cohort study to evaluate medication use in patients hospitalised with COVID-19 in Scotland: protocol for a national observational study. BMJ Open. 2021 Nov 1;11(11):e054861.

25. Scottish Government. The Scottish Index of Multiple Deprivation [Internet]. Statistics. 2020 [cited 2020 Oct 15]. Available from: https://www2.gov.scot/SIMD

26. Quan H, Sundararajan V, Halfon P, Fong A, Burnand B, Luthi JC, et al. Coding algorithms for defining comorbidities in ICD-9-CM and ICD-10 administrative data. Med Care. 2005 Nov;43(11):1130–9.

27. DataLab. Prescribing Data: BNF Codes [Internet]. 2017 [cited 2022 Feb 16]. Available from: https://www.thedatalab.org/blog/2017/04/prescribing-data-bnf-codes/

28. von Elm E, Altman DG, Egger M, Pocock SJ, Gøtzsche PC, Vandenbroucke JP. Strengthening the reporting of observational studies in epidemiology (STROBE) statement: guidelines for reporting observational studies. BMJ. 2007 Oct 18;335(7624):806–8.

29. UK Government. Cases in the UK | Coronavirus in the UK [Internet]. 2022 [cited 2022 Mar 4]. Available from: https://coronavirus.data.gov.uk/details/cases

30. Närhi F, Moonesinghe SR, Shenkin SD, Drake TM, Mulholland RH, Donegan C, et al. Implementation of corticosteroids in treatment of COVID-19 in the ISARIC WHO Clinical Characterisation Protocol UK: prospective, cohort study. The Lancet Digital Health. 2022 Apr 1;4(4):e220–34.

31. Amer M, Kamel AM, Bawazeer M, Maghrabi K, Butt A, Dahhan T, et al. Clinical characteristics and outcomes of critically ill mechanically ventilated COVID-19 patients receiving interleukin-6 receptor antagonists and corticosteroid therapy: a preliminary report from a multinational registry. European Journal of Medical Research. 2021 Oct 2;26(1):117.

32. Mustafa ZU, Kow CS, Salman M, Kanwal M, Riaz MB, Parveen S, et al. Pattern of medication utilization in hospitalized patients with COVID-19 in three District Headquarters Hospitals in the Punjab province of Pakistan. Exploratory Research in Clinical and Social Pharmacy. 2022 Mar 1;5:100101.

For peer review only - http://bmjopen.bmj.com/site/about/guidelines.xhtml

33. Ayodele O, Ren K, Zhao J, Signorovitch J, Funk MJ, Zhu J, et al. Real-world treatment patterns and clinical outcomes for inpatients with COVID-19 in the US from September 2020 to February 2021. PLOS ONE. 2021 Dec 28;16(12):e0261707.

34. Scottish Government. COVID Therapeutic Alert - Remdesivir for patients hospitalised due to COVID-19 (adults and adolescents 12 years and older) [Internet]. 2022 [cited 2022 Sep 29]. Available from: https://www.sehd.scot.nhs.uk/cmo/CEM_CMO(2022)006.pdf

35. Freeman A, Watson A, O'Regan P, Wysocki O, Burke H, Freitas A, et al. Wave comparisons of clinical characteristics and outcomes of COVID-19 admissions - Exploring the impact of treatment and strain dynamics. Journal of Clinical Virology. 2022 Jan 1;146:105031.

36. Wong CKH, Lau KTK, Au ICH, Xiong X, Lau EHY, Cowling BJ. Clinical Improvement, Outcomes, Antiviral Activity, and Costs Associated With Early Treatment With Remdesivir for Patients With Coronavirus Disease 2019 (COVID-19). Clinical Infectious Diseases. 2021 Jul 15;ciab631.

37. Clift AK, Coupland CAC, Keogh RH, Diaz-Ordaz K, Williamson E, Harrison EM, et al. Living risk prediction algorithm (QCOVID) for risk of hospital admission and mortality from coronavirus 19 in adults: national derivation and validation cohort study. BMJ. 2020 Oct 20;371:m3731.

38. SPICe. Timeline of Coronavirus (COVID-19) in Scotland [Internet]. SPICe Spotlight | Solas air SPICe. 2022 [cited 2022 Feb 15]. Available from: https://spice-spotlight.scot/2022/02/11/timeline-of-coronavirus-covid-19-in-scotland/

Figure legends

Figure 1: Prescribing of drugs of interest over time in Scotland, August 2020 to October 2021

Figure 2: COVID-19 hospitalisations in Scotland, June 2020 to November 2021

For peer review only

Figure 1

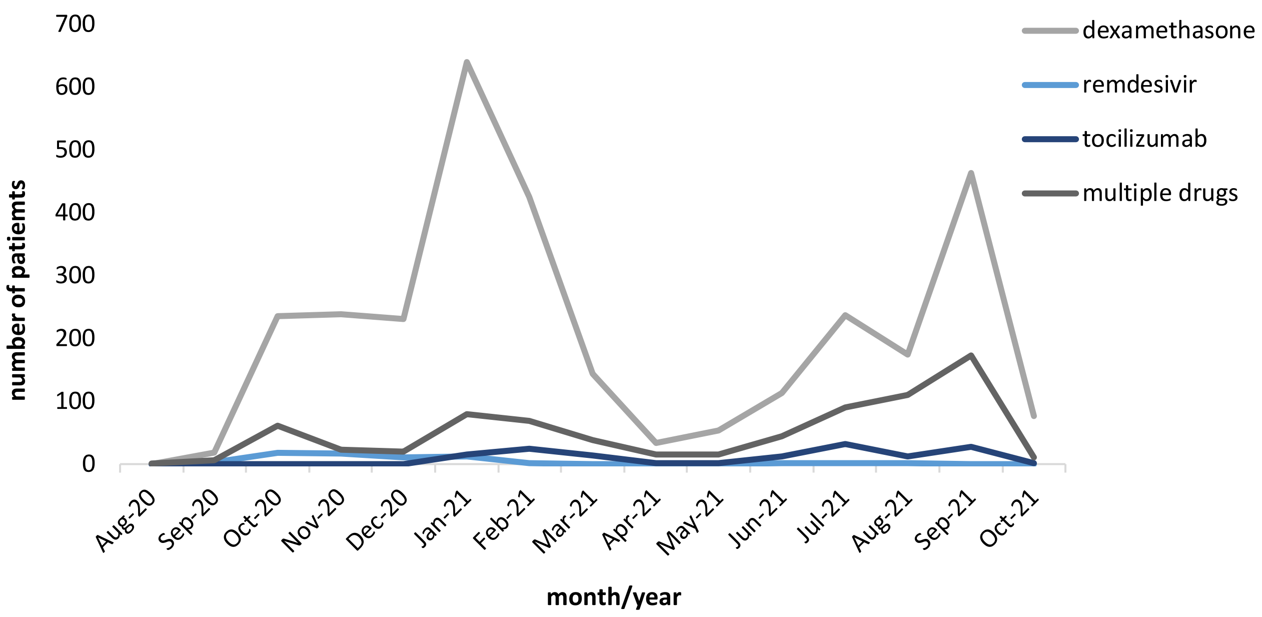

Figure 1: Prescribing of drugs of interest over time in Scotland, August 2020 to October 2021

Figure 2

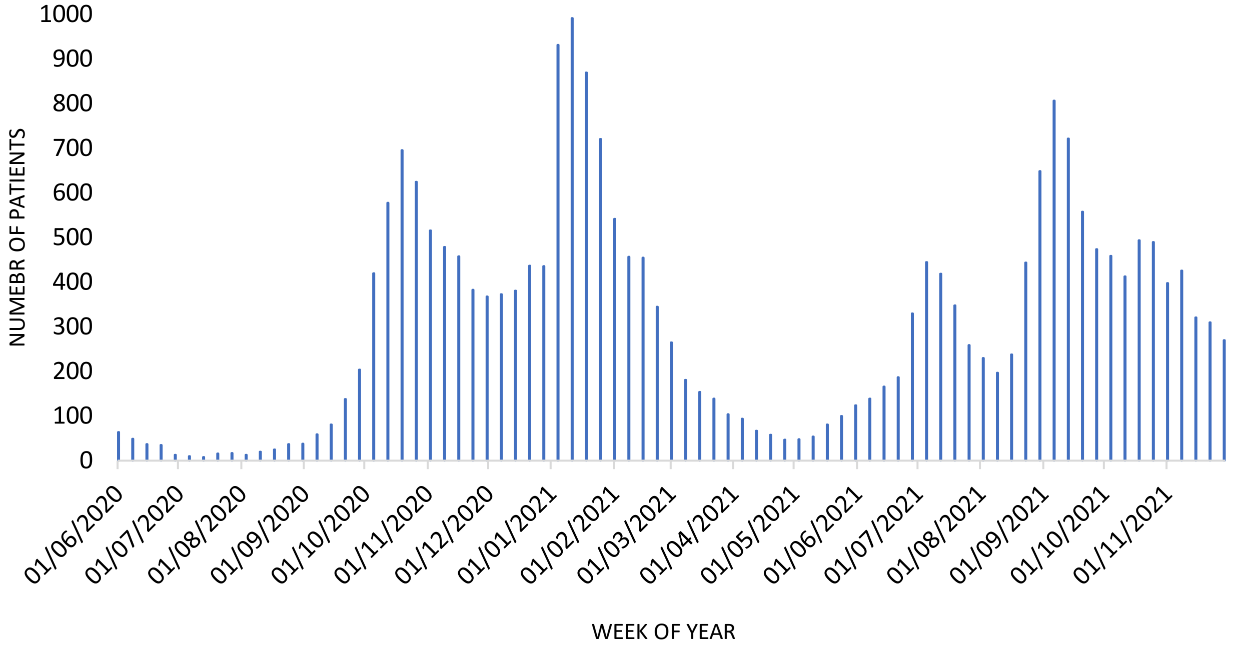

Figure 2: COVID-19 hospitalisations in Scotland, June 2020 to November 2021

Supplementary material

Table S1: Patient baseline characteristics, monotherapy by individual drug, Scotland

n (%)	Dexamethasone n=3,094	Remdesivir n=68	Tocilizumab n=145
Socio-demographics			
Median age (IQR) [years]	66 (54 – 78)	65.5 (55 – 74)	57 (45 – 66)
Age 18 – 40 [years]	342 (11.1)	5 (7.4)	31 (21.4)
Age 41 – 70 [years]	1,475 (47.7)	39 (57.4)	87 (60.0)
Age > 70 [years]	1,277 (41.3)	24 (35.3)	27 (18.6)
Sex [male]	1,659 (53.6)	40 (58.8)	91 (62.8)
Most deprived [quintile][1]	927 (30.0)	31 (45.6)	42 (29.0)
Least deprived [quintile][1]	280 (9.0)	8 (11.8)	14 (9.7)
Region: Health Board			
Ayrshire & Arran	779 (25.2)	2 (2.9)	23 (15.9)
Dumfries & Galloway	156 (5.0)	4 (5.9)	5 (3.4)
Forth Valley	656 (21.2)	1 (1.5)	19 (13.1)
Greater Glasgow & Clyde	461 (14.9)	6 (8.8)	13 (9.0)
Lanarkshire	906 (29.3)	53 (77.9)	76 (52.4)
Lothian	136 (4.4)	2 (2.9)	9 (6.2)
Vaccination status			
Unvaccinated	2,316 (74.9)	63 (92.6)	94 (64.8)
1 dose	125 (4.0)	1 (1.5)	40 (27.6)
2 doses	653 (21.1)	4 (5.9)	11 (7.6)
**Charlson score**[2]			
0	1,570 (50.7)	35 (51.5)	90 (62.1)
1 – 2	1,028 (33.2)	23 (33.8)	47 (32.4)
3 – 4	343 (11.1)	6 (8.8)	4 (2.8)
> 4	153 (4.9)	4 (5.9)	4 (2.8)
Medication at baseline: number of different items[3]			
0	294 (9.5)	4 (5.9)	34 (23.5)
1 – 4	613 (19.2)	15 (22.1)	43 (29.7)
5 – 10	1,041 (33.7)	21 (30.9)	38 (26.2)
> 10	1,146 (37.0)	28 (41.2)	30 (20.7)
Medication at baseline: specific drug classes [yes][3]			
Antihypertensive drugs	1,671 (54.0)	41 (60.3)	51 (35.2)
Antithrombotic drugs	1,093 (35.3)	22 (32.3)	36 (24.8)
Statins	1,187 (38.4)	31 (45.6)	34 (23.5)
Bronchodilator	793 (25.6)	17 (25.0)	25 (17.2)
Inhaled steroid	643 (20.8)	16 (23.5)	23 (15.9)
Diabetes medication	607 (19.6)	10 (14.7)	21 (14.5)

[1] Deprivation based on the Scottish Index of Multiple Deprivation quintiles, where 1=most deprived and 5=least deprived (25)

[2] Charlson score at baseline based on diagnoses from hospital discharge records during the 5-year period directly preceding the admission, identified using ICD-10 codes (26)

[3] Polypharmacy and medication at baseline based on prescriptions dispensed in community pharmacy during the 6-months period directly preceding the admission, identified using BNF codes (27); includes both acute and chronic medication

For peer review only

Table S2: Patient baseline characteristics, combination therapy, Scotland

n (%)	Dexamethasone & remdesivir n=185	Dexamethasone & tocilizumab n=530	Remdesivir & tocilizumab n=10	Dexamethasone, remdesivir & tocilizumab n=31
Socio-demographics				
Median age (IQR) [years]	65 (52 – 73.5)	58 (45 – 70)	66 (60 – 74)	56 (46 – 65)
Age 18 – 40 [years]	21 (11.4)	100 (18.8)	0	5 (16.1)
Age 41 – 70 [years]	101 (54.9)	303 (57.1)	6 (60.0)	22 (71.0)
Age > 70 [years]	62 (33.7)	128 (24.1)	4 (40.0)	4 (12.9)
Sex [male]	107 (58.2)	336 (63.3)	7 (70.0)	20 (64.5)
Most deprived [quintile][1]	57 (31.0)	156 (29.4)	3 (30.0)	11 (35.5)
Least deprived [quintile][1]	18 (9.8)	58 (10.9)	0	5 (16.3)
Region: Health Board				
Ayrshire & Arran	5 (2.7)	59 (11.1)	0	0
Dumfries & Galloway	17 (9.2)	62 (11.7)	1 (10.0)	3 (9.7)
Forth Valley	22 (12.0)	60 (11.3)	0	0
GGC	43 (23.4)	72 (13.6)	4 (40.0)	15 (48.4)
Lanarkshire	85 (46.2)	224 (42.2)	4 (40.0)	5 (16.1)
Lothian	12 (6.5)	54 (10.2)	1 (10.0)	8 (25.8)
Vaccination status				
Unvaccinated	148 (80.4)	321 (60.5)	7 (70.0)	15 (48.4)
1 dose	3 (1.6)	28 (5.3)	0	4 (12.9)
2 doses	33 (17.9)	182 (34.3)	3 (30.0)	12 (38.7)
**Charlson score**[2]				
0	96 (51.9)	348 (65.7)	7 (70.0)	13 (41.9)
1 – 2	69 (37.5)	156 (29.4)	2 (20.0)	14 (45.2)
3 – 4	12 (6.5)	17 (3.3)	0	1 (3.2)
>4	8 (4.4)	9 (1.7)	1 (10.0)	3 (9.7)
Medication at baseline: number of different items[3]				
0	15 (8.2)	97 (19.3)	2 (20.0)	1 (3.2)
1 – 4	40 (21.7)	141 (26.6)	3 (30.0)	9 (29.0)
5 – 10	60 (32.6)	162 (30.5)	3 (30.0)	14 (45.2)
> 10	69 (37.5)	131 (24.7)	2 (20.0)	7 (22.6)
Medication at baseline: specific drug classes [yes][3]				
Antihypertensive drugs	103 (56.0)	248 (46.7)	5 (50.0)	13 (41.9)
Antithrombotic drugs	57 (31.0)	129 (24.3)	4 (40.0)	7 (22.6)
Statins	74 (40.2)	173 (32.6)	4 (40.0)	8 (25.8)
Bronchodilator	57 (31.0)	85 (16.0)	2 (20.0)	5 (16.1)
Inhaled steroid	44 (23.9)	80 (15.1)	2 (20.0)	6 (19.4)
Diabetes medication	35 (19.0)	98 (18.5)	3 (30.0)	4 (12.9)

[1] Deprivation based on the Scottish Index of Multiple Deprivation quintiles, where 1=most deprived and 5=least deprived (25)

[2] Charlson score at baseline based on diagnoses from hospital discharge records during the 5-year period directly preceding the admission, identified using ICD-10 codes (26)

[3] Polypharmacy and medication at baseline based on prescriptions dispensed in community pharmacy during the 6-months period directly preceding the admission, identified using BNF codes (27); includes both acute and chronic medication

For peer review only

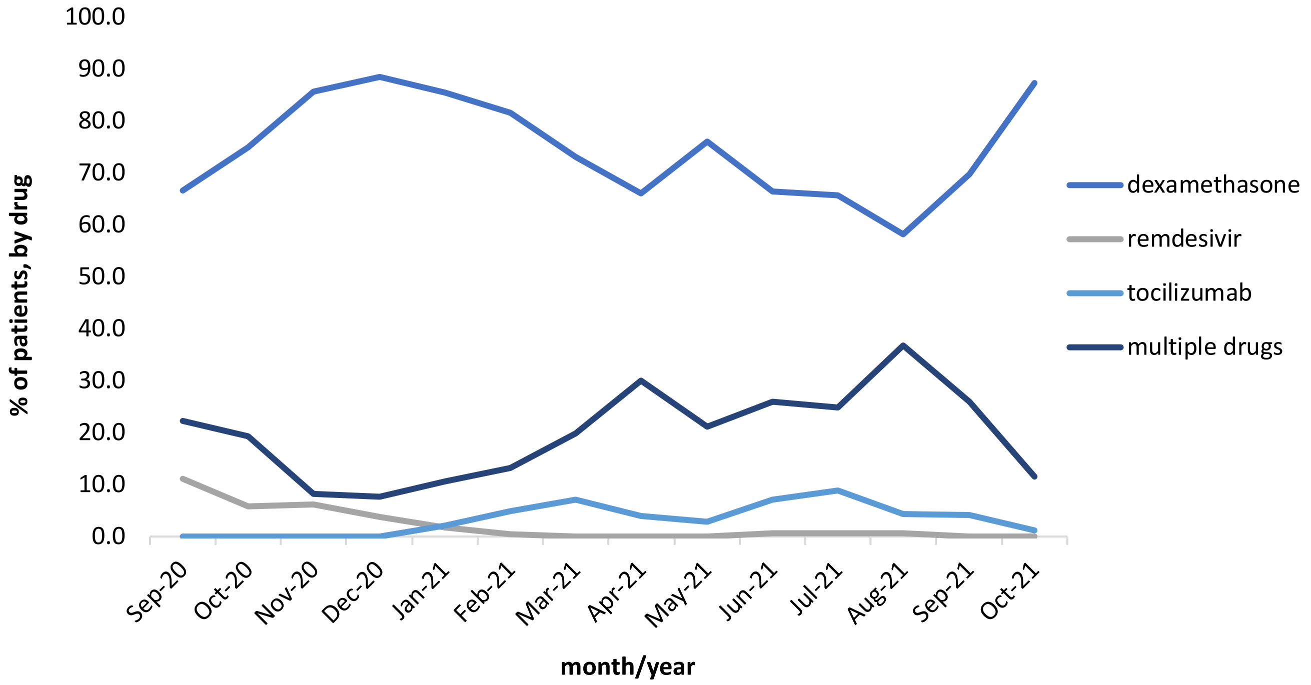

Figure S1: Percentage of patients on each treatment option over time, by month

STROBE Statement—checklist of items that should be included in reports of observational studies (**Assessing medication use in COVID-19**)

	Item No.	Recommendation	Page No.
**Title and abstract**	1	(*a*) Indicate the study's design with a commonly used term in the title or the abstract	Title page
		(*b*) Provide in the abstract an informative and balanced summary of what was done and what was found	1
**Introduction**			
Background/rationale	2	Explain the scientific background and rationale for the investigation being reported	3,4
Objectives	3	State specific objectives, including any prespecified hypotheses	4
**Methods**			
Study design	4	Present key elements of study design early in the paper	4
Setting	5	Describe the setting, locations, and relevant dates, including periods of recruitment, exposure, follow-up, and data collection	4-6
Participants	6	(*a*) *Cohort study*—Give the eligibility criteria, and the sources and methods of selection of participants. Describe methods of follow-up<br>*Case-control study*—Give the eligibility criteria, and the sources and methods of case ascertainment and control selection. Give the rationale for the choice of cases and controls<br>*Cross-sectional study*—Give the eligibility criteria, and the sources and methods of selection of participants	5,6
		(*b*) *Cohort study*—For matched studies, give matching criteria and number of exposed and unexposed<br>*Case-control study*—For matched studies, give matching criteria and the number of controls per case	n/a (no matching)
Variables	7	Clearly define all outcomes, exposures, predictors, potential confounders, and effect modifiers. Give diagnostic criteria, if applicable	6
Data sources/ measurement	8*	For each variable of interest, give sources of data and details of methods of assessment (measurement). Describe comparability of assessment methods if there is more than one group	5
Bias	9	Describe any efforts to address potential sources of bias	n/a (purely descriptive study)
Study size	10	Explain how the study size was arrived at	n/a (all available patients)

Continued on next page

For peer review only - http://bmjopen.bmj.com/site/about/guidelines.xhtml

Quantitative variables	11	Explain how quantitative variables were handled in the analyses. If applicable, describe which groupings were chosen and why	6
Statistical methods	12	(*a*) Describe all statistical methods, including those used to control for confounding	6
		(*b*) Describe any methods used to examine subgroups and interactions	n/a (purely descriptive study)
		(*c*) Explain how missing data were addressed	n/a (purely descriptive study)
		(*d*) *Cohort study*—If applicable, explain how loss to follow-up was addressed<br>*Case-control study*—If applicable, explain how matching of cases and controls was addressed<br>*Cross-sectional study*—If applicable, describe analytical methods taking account of sampling strategy	n/a
		(*e*) Describe any sensitivity analyses	n/a (purely descriptive study)
**Results**			
Participants	13*	(a) Report numbers of individuals at each stage of study—eg numbers potentially eligible, examined for eligibility, confirmed eligible, included in the study, completing follow-up, and analysed	7 (all eligible patients have been included in descriptive analyses)
		(b) Give reasons for non-participation at each stage	n/a
		(c) Consider use of a flow diagram	
Descriptive data	14*	(a) Give characteristics of study participants (eg demographic, clinical, social) and information on exposures and potential confounders	8
		(b) Indicate number of participants with missing data for each variable of interest	n/a (available data on the exposure was the outcome of interest)
		(c) *Cohort study*—Summarise follow-up time (eg, average and total amount)	n/a
Outcome data	15*	*Cohort study*—Report numbers of outcome events or summary measures over time	8-10
		Case-control study—Report numbers in each exposure category, or summary measures of exposure	
		Cross-sectional study—Report numbers of outcome events or summary measures	
Main results	16	(*a*) Give unadjusted estimates and, if applicable, confounder-adjusted estimates and their precision (eg, 95% confidence interval). Make clear which confounders were adjusted for and why they were included	n/a (purely descriptive study)
		(*b*) Report category boundaries when continuous variables were categorized	n/a
		(*c*) If relevant, consider translating estimates of relative risk into absolute risk for a meaningful time period	n/a

Continued on next page

For peer review only - http://bmjopen.bmj.com/site/about/guidelines.xhtml

Other analyses	17	Report other analyses done—eg analyses of subgroups and interactions, and sensitivity analyses	n/a
**Discussion**			
Key results	18	Summarise key results with reference to study objectives	10
Limitations	19	Discuss limitations of the study, taking into account sources of potential bias or imprecision. Discuss both direction and magnitude of any potential bias	13
Interpretation	20	Give a cautious overall interpretation of results considering objectives, limitations, multiplicity of analyses, results from similar studies, and other relevant evidence	10-12
Generalisability	21	Discuss the generalisability (external validity) of the study results	12,13
**Other information**			
Funding	22	Give the source of funding and the role of the funders for the present study and, if applicable, for the original study on which the present article is based	14

*Give information separately for cases and controls in case-control studies and, if applicable, for exposed and unexposed groups in cohort and cross-sectional studies.

Note: An Explanation and Elaboration article discusses each checklist item and gives methodological background and published examples of transparent reporting. The STROBE checklist is best used in conjunction with this article (freely available on the Web sites of PLoS Medicine at http://www.plosmedicine.org/, Annals of Internal Medicine at http://www.annals.org/, and Epidemiology at http://www.epidem.com/). Information on the STROBE Initiative is available at www.strobe-statement.org.

For peer review only
